# N-acetylaspartate promotes glycolytic-to-oxidative fiber-type switch and resistance to atrophic stimuli in myotubes

**DOI:** 10.1038/s41419-024-07047-0

**Published:** 2024-09-19

**Authors:** Serena Castelli, Enrico Desideri, Leonardo Laureti, Federica Felice, Angela De Cristofaro, Silvia Scaricamazza, Giacomo Lazzarino, Maria Rosa Ciriolo, Fabio Ciccarone

**Affiliations:** 1 IRCCS San Raffaele Roma, Rome, Italy; 2 Department of Human Sciences and Promotion of the Quality of Life, San Raffaele Open University, Rome, Italy; 3 Department of Biology, University of Rome Tor Vergata, Rome, Italy; 4 IRCCS Fondazione Santa Lucia, Rome, Italy; 5 UniCamillus-Saint Camillus International University of Health and Medical Sciences, Rome, Italy

**Keywords:** Biochemistry, Neuroscience

## Abstract

N-acetylaspartate (NAA) is a neuronal metabolite that can be extruded in extracellular fluids and whose blood concentration increases in several neurodegenerative disorders, including amyotrophic lateral sclerosis (ALS). Aspartoacylase (ASPA) is the enzyme responsible for NAA breakdown. It is abundantly expressed in skeletal muscle and most other human tissues, but the role of NAA catabolism in the periphery is largely neglected. Here we demonstrate that NAA treatment of differentiated C2C12 muscle cells increases lipid turnover, mitochondrial biogenesis and oxidative metabolism at the expense of glycolysis. These effects were ascribed to NAA catabolism, as CRISPR/Cas9 ASPA KO cells are insensitive to NAA administration. Moreover, the metabolic switch induced by NAA was associated with an augmented resistance to atrophic stimuli. Consistently with in vitro results, SOD1-G93A ALS mice show an increase in ASPA levels in those muscles undergoing the glycolytic to oxidative switch during the disease course. The impact of NAA on the metabolism and resistance capability of myotubes supports a role for this metabolite in the phenotypical adaptations of skeletal muscle in neuromuscular disorders.

## Introduction

N-acetylaspartate (NAA) is the second most abundant metabolite in the central nervous system (CNS) reaching concentrations of approximately 10 mM or greater. NAA is synthesized from aspartate and acetyl-CoA by the mitochondrial enzyme N-acetyltransferase 8 like (NAT8L), whereas the catabolism is due to aspartoacylase (ASPA) enzyme activity. In the CNS, NAA synthesis mainly occurs in neurons, which accumulate it due to the almost complete lack of ASPA [1]. NAA can be released in the CNS extracellular space and promptly taken up by glial cells, particularly oligodendrocytes, which express ASPA enzyme and can utilize NAA as a source of acetyl-CoA for fatty acid synthesis and acetylation processes [2]. Given that NAA is predominantly stored inside neuronal cells, NAA concentration in cerebrospinal fluid and blood is generally low in healthy subjects, but it significantly increases in neurodegenerative diseases [3]. Despite the ability of NAA to reach any district of the body via the circulatory system, few studies have focused on the role of the NAA pathway in peripheral tissues. Among them, NAA metabolism in tissues other than the brain has been assessed in brown adipose tissue (BAT). Brown adipocytes express NAT8L enzyme at levels almost comparable to those of neurons and, expressing also ASPA enzyme, they can simultaneously break down NAA [4, 5]. In this tissue, NAA synthesis and catabolism are coupled with an increase in lipogenesis and lipolysis, determining a “futile” cycle that continuously supports mitochondrial oxidation and thermogenesis [6, 7]. Apart from neurons and brown adipocytes, NAT8L expression is very weak in other organs while ASPA expression is more widely distributed in all peripheral tissues, including skeletal muscle, suggesting that NAA catabolism has functional consequences in the periphery.

Skeletal muscle, the most abundant tissue of the human body, displays heterogeneity in myofibers that depends on the speed of contraction and the energetic fuel they use. Myofibers can be divided into two major types, type I (or slow-twitch) fibers and type II (or fast-twitch) fibers. Type II fibers can be further divided into the subtypes IIA and IIB/X [8]. The slow-twitch type I myofibers have a slow speed of contraction and are the most fatigue-resistant. They are highly vascularized and show high levels of mitochondria and myoglobin, and mostly rely on lipid catabolism and oxidative phosphorylation for energy production. The fast-twitch type IIB/X fibers are characterized by a rapid contraction of the sarcomere, are easily fatigued, and mainly depend on glycolysis for ATP production; the type IIA fibers exhibit an intermediate behavior and metabolism between slow-twitch type I fibers and highly glycolytic type IIB/X fibers. The relative abundance of each type of fibers in muscles is influenced by several factors like lifestyle, training, age and pathological conditions [6]. Notably, glycolytic-to-oxidative fiber-type switch has been observed in motor neuron diseases such as amyotrophic lateral sclerosis (ALS), where muscles undergo an increase in lipid oxidative metabolism and replace type-IIB myosin heavy chain (MHC) isoforms with type IIA ones [9, 10].

Considering the metabolic alterations occurring in ALS muscle, the fact that serum NAA levels increase in ALS [11] and that the degeneration of spinal motor neurons may locally increase NAA concentration, we aimed to characterize the contribution of NAA in the rewiring of muscle cell metabolism. We analyzed the effects of NAA treatment in an in vitro model of skeletal muscle, namely differentiated C2C12 myotubes. Metabolic consequences of NAA administration were coupled with analysis of myotube morphology, identity, and response to atrophic stimuli. Evidence of a key role of NAA catabolism on muscle metabolism was provided by using ASPA CRISPR/Cas9 knock-out (KO) C2C12 cells, published datasets and muscle biopsies from the SOD1-G93A ALS mouse model.

## Materials and methods

### Materials

Materials used for this work are detailed in the Supplementary Materials.

### Cell lines, treatments and animals

The cell line of murine myoblasts C2C12 was purchased from the American Type Culture Collection (ATCC). Cells were grown in Dulbecco’s modified Eagle’s medium (DMEM) 4.5 g/L glucose, supplemented with 10% fetal bovine serum, 10 U/ml penicillin/streptomycin, 2 mM L-glutamine, 1 mM sodium pyruvate and 10 mM HEPES pH 7.0. Cells were cultured at 37 °C in an atmosphere of 5% CO_2_ in the air and, for all the experiments, they were plated at a 5 × 10^4^ cells/mL density on wells covered by 0.1 g/mL of gelatin derived from bovine skin (G9391, Sigma Aldrich). After 24 h, C2C12 differentiation in myotubes was induced by a specific differentiation medium consisting of DMEM containing 4.5 g/L of glucose, supplemented with 2% of horse serum (HS), 2 mM of L-glutamine, 10 U/mL penicillin/streptomycin solution, 1 mM sodium pyruvate and 10 mM HEPES pH 7.0. The differentiation was induced for 7 days, changing the medium every day, for all experiments, except where differently indicated. The treatment with NAA (Sigma Aldrich, 00920) was added after 5 days of differentiation and was left for 48 h, until the end of differentiation.

SOD1-G93A mice (B6.Cg-Tg(SOD1 G93A)1Gur/J) were purchased from The Jackson Laboratory (Bar Harbor, ME, USA). Hemizygous SOD1-G93A males and C57BL/6 females were crossbred, and progeny were genotyped by PCR. Disease onset was evaluated by hanging grid test; 20–22 weeks-old mice were defined as “symptomatic”. Before sacrifice, mice were anaesthetized with Rompum (xylazine, 20 mg/ml, 0.5 ml/kg Bayer, Milan, Italy) plus Zoletil (tiletamine and zolazepam, 100 mg/ml, 0.5 ml/kg; Virbac, Milan, Italy). A detailed description of treatments and animals’ management was provided in Supplementary Materials.

### Knock-out production by CRISPR/Cas9 technology

The generation of ASPA and ATGL Knock-Out (KO) C2C12 cells was performed by the CRISPR/Cas9 technology. For ASPA KO, the Cas9 enzyme was directed by gRNA, obtained from the following guides: ASPA 1, forward 5'-CACCGAGTGCAACCCATGTTAGAAG-3' and reverse 5'-AAACCTTCTAACATGGGTTGCACTC-3'; ASPA 2 forward 5'-CACCGAGTGCAACCCATGTTAGAAG-3' and reverse 5'-AAACCTTCTAACATGGGTTGCACTC-3'. For ATGL KO, the following guides were used: ATGL 1, forward 5'-CACCGAGAGGCGGTAGAGATTGCGA-3' and reverse 5'-AAACTCGCAATCTCTACCGCCTCTC-3'; ATGL 2, forward 5'-CACCGGCAGGAGGCCACGCCAATG-3' and reverse 5'-AAACCATTGGCGTGGCCTCCTGCC-3'. For control, only the Cas9 plasmid was transfected. The detailed construction was provided in Supplementary information.

### Western blot analysis

Detailed methods and antibodies were provided in Supplementary information.

### Fluorescence microscopy analyses

Live cells were treated with 2.5 µM JC-1 (420200, Calbiochem) for 30 min at 37 °C. Delta Vision Restoration Microscopy System (Applied Precision, Issaquah, WA) equipped with an Olympus IX70 fluorescence microscope (Olympus Italia, Segrate, Milano, Italy) was used to acquire fluorescent images of cells. Detailed methods were provided in Supplementary information.

### Extracellular lactate assay

Cell medium was precipitated with 30% trichloroacetic acid (TCA). Media were stored at −20 °C for at least 1 h and then centrifuged at 14,000 × *g* for 20 min at 4 °C. Supernatant was incubated with reaction buffer containing LDH enzyme for 30 min at 37 °C. The amount of lactate was quantified by measuring NADH absorbance at 340 nm using an Eppendorf BioSpectrometer. Concentrations were normalized on total proteins in each sample. The detailed protocol was provided in Supplementary Materials.

### ATP quantification assay

ATP quantification was performed by using ATP Assay Kit - Colorimetric Kit (Assaygenie). The assay protocol relies on the production of creatine phosphate from creatine and ATP. The content of phosphocreatine is detected by colorimetric method to reflect ATP content.

### Oil Red-O staining

Cells were fixed in formalin solution neutral buffered 10% and, after PBS washes, were incubated for 5 min with 60% isopropanol and stained with the Oil Red-O for 10 min. Oil Red-O was removed and di-deionized water was used to wash cells. Delta Vision Restoration Microscopy System (Applied Precision) equipped with an Olympus IX70 fluorescence microscope (Olympus) was used to acquire fluorescent images of cells. Detailed methods were provided in Supplementary information.

### Complex I activity assay

The activity of the NADH:ubiquinone oxidoreductase complex I was measured through a direct method [12], following the decrease in absorbance at 340 nm due to the oxidation of NADH. A detailed description of this protocol is provided in Supplementary Materials.

### RNA isolation and real-time qPCR

RNA extraction was performed by using TRItidy G (PanReac AppliChem). Synthesis of cDNA was obtained from 1 μg of total RNA by using iScript™ Reverse Transcription Supermix for RT-qPCR (Bio-Rad), and RT-qPCR reaction was performed by using the iTaq Universal SYBR Green Supermix (Bio-Rad) on QuantStudio™ 3 real-time PCR System (Thermo Fisher Scientific). The list of primers is contained in Supplementary Materials.

### HPLC analysis

For HPLC analysis, details were provided in Supplementary Materials.

### Bioinformatic analyses

The detailed list of databases used was provided in Supplementary Materials.

## Results

### NAA promotes lipid turnover and an oxidative phenotype in C2C12 myotubes

**Fig. 1:**
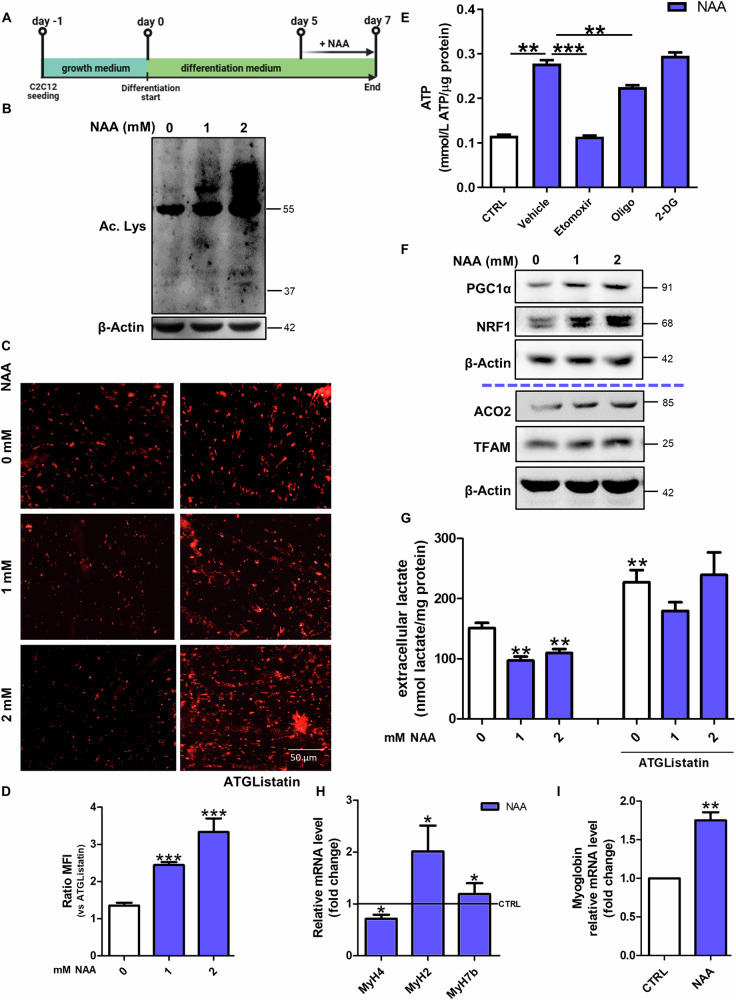
NAA promotes lipid-dependent oxidative metabolism in myotubes. **A** Graphical summary of the experimental design. **B** Western blot analysis of acetyl-lysine levels. β-Actin was used as a loading control. **C** Representative images of differentiated C2C12 myotubes after staining with Oil Red-O. Scale bars, 50 µm. (n = 3) **D** The ratio of MFI of untreated and treated cells with ATGListatin. Data are expressed as mean ± SD of n = 3 independent experiments (***p < 0.001 vs untreated). **E** Colorimetric quantification of ATP after 2 mM NAA treatment and co-treatment with oligomycin (2 µM), 2-deoxyglucose (40 mM) and etomoxir (20 µM). Data are expressed as mmol of ATP produced per µg of protein. (** p < 0.01¸ *** *p* < 0.001). **F** Western blot analysis of PGC1α, NRF1, ACO2 and TFAM levels on C2C12 myotubes. β-Actin was used as loading control. The Western blots reported are representative of three independent experiments that gave similar results. **G** Evaluation of extracellular lactate content after NAA co-treatment with ATGListatin. Lactate concentration was normalized on total proteins and data are expressed as mean ± SD of n = 3 independent experiments (** p < 0.01 vs 0 mM). RT-qPCR analysis of **H** *MyH4, MyH2*, *MyH7b* and **I** *myoglobin* in C2C12 myotubes. *ACTB* was used as a reference control. Data are shown as fold change ± SD of n = 3 independent experiments (*p < 0.05, **p < 0.01 vs CTRL). CTRL was represented by a straight line in the bar graph.

To identify the effects of NAA on the metabolism of diffeentiated C2C12 myotubes, NAA was administered at day 5 of differentiation and maintained for 48 h (Fig. 1A). At that time, cells showed the highest expression of the late differentiation marker MHC (Supplementary Fig. 1A, B) and myotubes were already formed (Supplementary Fig. 1C).

NAA is known to provide acetyl-CoA for acetylation processes and fatty acid synthesis [13], thus we examined acetyl-lysine levels and lipid droplets (LDs) content. As expected, acetyl-lysine levels increased after NAA treatment (Fig. 1B; Supplementary Fig. 1D). Although Oil Red-O staining showed a decrease in LDs content after NAA administration, in myotubes co-treated with the lipolytic inhibitor ATGListatin or knock-out (KO) for Adipose triglyceride lipase (ATGL) the accumulation of LDs was higher than control cells (Fig. 1C, D; Supplementary Fig. 1E-H). This result is compatible with a boosted lipid synthesis and degradation in these cells. The increased lipogenesis was further confirmed by the dephosphorylation/activation of Acetyl-CoA carboxylase1 (ACC1) (Supplementary Fig. 1I, J) while the increase in lipid catabolism following NAA administration was confirmed by the decrement of Oil Red-O staining in cells pre-treated with oleic acid (Supplementary Fig. 1K), which was used for boosting LDs accumulation, and by the upregulation of the lipases ATGL and HSL (Supplementary Fig. 1L, M). Confirmatory results of the effect of NAA on LDs turnover were obtained both with higher concentrations of NAA (Supplementary Fig. 2A, B), and when NAA treatment was performed on C2C12 cells differentiated for 15 days (Supplementary Fig. 2C, D).

This augmentation in lipid catabolism was also corroborated by the increase in ATP production after NAA treatment, which was dramatically abrogated by blocking mitochondrial fatty acid import with etomoxir (Cpt1α inhibitor). This results indicates that the increase in ATP levels after NAA treatment is mainly due to oxidative phosphorylation fueled by fatty acid oxidation (Fig. 1E) as confirmed by the decreased ATP concentration observed after oligomycin (ATP synthase inhibitor) treatment and not after inhibition of glycolysis by 2-deoxyglucose (2-DG). Coherently, mitochondrial mass was also augmented by NAA, as shown by the increase of ACO2 and TFAM levels, thanks to a prompted mitochondrial biogenesis, as suggested by higher levels of PGC1α and NRF1 (Fig. 1F; Supplementary Fig. 2E). The increase in mitochondrial membrane potential revealed by the increased ratio between the red and green fluorescence of the JC-1 dye (Supplementary Fig. 2F, G) and the high activity of mitochondrial complex I clarified that mitochondria are more active after NAA treatment (Supplementary Fig. 2H). This increase in mitochondrial oxidative metabolism was paralleled by a decrease in the glycolytic rate as demonstrated by the reduction of the extracellular lactate extruded by NAA-treated C2C12 myotubes (Fig. 1G). Notably, this metabolic shift was likely due to the higher lipid usage induced by NAA, as blocking lipid catabolism by ATGListatin did not alter extracellular lactate levels in NAA-treated cells (Fig. 1G). As myofiber identity corresponds to a specific metabolic setting [6, 14], the markers of the three types of myofibers (slow- intermediate- and fast-twitch) were evaluated after NAA treatment. MHC IIb (encoded by *MyH4* gene), typically expressed in fast glycolytic fibers, was decreased, whereas the fast oxidative/intermediate and slow oxidative markers MHC IIa (encoded by *MyH2* gene) and MHC7b (encoded by *MyH7b* gene) were increased by NAA (Fig. 1H). These results demonstrate that NAA elicits a glycolytic-to-oxidative fiber-type switch in muscle cells, as further confirmed by the upregulation of myoglobin (Fig. 1I), which supports oxidative phosphorylation in oxidative myofibers.

### NAA decreases myofiber diameter by enhancing protein turnover

**Fig. 2:**
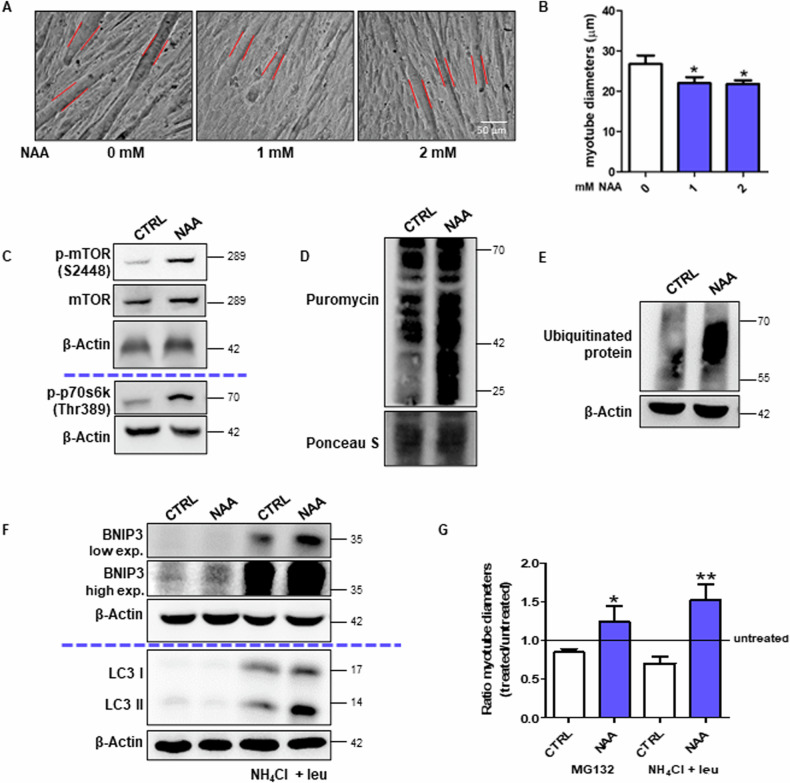
NAA promotes protein turnover affecting myotube diameter. **A** Bright-field images of C2C12 myotubes. Scale bars, 50 µm. (n = 3). **B** The diameter measurement performed by ImageJ software. Data are expressed as mean ± SD of n = 3 independent experiments (*p < 0.05 vs 0 mM). **C** Western blot analysis of p-mTOR, mTOR and p-p70s6k (Thr389) levels in C2C12 myotubes. β-Actin was used as a loading control. **D** Western blot analysis of puromycin incorporation levels. Ponceau S was used as a loading control. Western blot analysis of **E** ubiquitinated protein levels and **F** BNIP3 and LC3 levels with/without NH_4_Cl and leupeptin. β-Actin was used as a loading control. The Western blots reported are representative of three independent experiments that gave similar results. **G** The diameter measurement of bright-field images of myotubes MG132 or NH_4_Cl/leupeptin treatment was performed by ImageJ software. Data are expressed as mean ± SD of n = 3 independent experiments (*p < 0.05, **p < 0.01 vs untreated).

In order to evaluate whether the molecular and metabolic signatures induced by NAA on C2C12 myotubes are associated with phenotypic changes in the fibers, we measured myotube diameters in brightfield images. We observed that NAA treatment reduced the diameter of myotubes (Fig. 2A, B), which is consistent with the acquisition of slow-twitch fiber phenotypes [8]. The different diameter between NAA-treated and untreated myotubes was not due to a discrepancy in the differentiation capability or the activation of atrophic response after NAA treatment, as demonstrated by the unchanged fusion index (Supplementary Fig. 3A) and the unaltered or even reduced levels of the main atrophic markers *TRIM63* (also known as Murf1) and *FBXO32* (also known as Atrogin1) (Supplementary Fig. 3B).

Thereafter, we evaluated the main pathways involved in protein synthesis and degradation that can promote a higher protein turnover rate, which is a feature of type I fibers and a process required for myofiber remodeling [15]. The higher phosphorylation levels of mTOR-S6K pathway components demonstrated a more active protein synthesis (Fig. 2C; Supplementary Fig. 3C, D) in C2C12 cells treated with NAA, as also confirmed by the puromycin incorporation assay (Fig. 2D, Supplementary Fig. 3E). The proteasomal degradation pathway was analyzed by monitoring the levels of ubiquitinated proteins, which resulted slightly increased after NAA treatment (Fig. 2E; Supplementary Fig. 3F). The high levels of BNIP3 and LC3-II in NAA-treated cells upon autophagy inhibition with NH_4_Cl/leupeptin demonstrated a massive activation of autophagy after NAA treatment (Fig. 2F; Supplementary Fig. 3G, H). Given the activation of both these degradation pathways upon NAA treatment, their role in myotube phenotypic changes was tested. By blocking either proteasome or autophagy, the reduction in myotube diameter was abrogated upon NAA (Fig. 2G, Supplementary Fig. 3I).

### The metabolic and phenotypical effects of NAA are determined by its catabolism

**Fig. 3:**
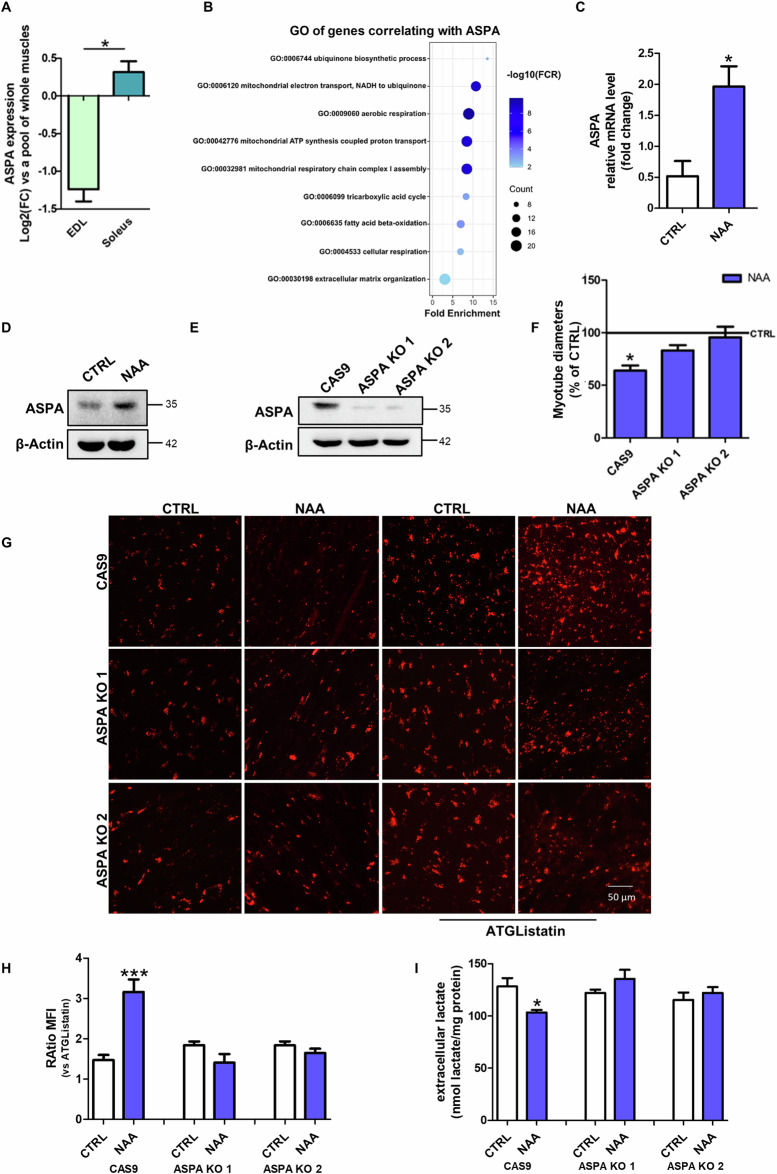
ASPA-mediated catabolism is responsible for NAA effects in mytubes. **A** Bioinformatic analysis of ASPA expression in EDL and soleus muscles. Data are expressed as Log_2_(fold change) ± SD (*p < 0.05 EDL vs soleus). **B** Bioinformatic analysis of GO of transcripts that correlate with ASPA expression in muscle. **C** RT-qPCR analysis of *ASPA* expression in C2C12 cells. *ACTB* was used as a reference control. Data are shown as fold change ± SD of n = 3 independent experiments (* *p* < 0.05 vs CTRL). Western blot analysis of ASPA levels on **D** C2C12 myotubes treated with NAA for 48 h and on **E** differentiated ASPA KO C2C12 cells. The Western blots reported are representative of three independent experiments that gave similar results. **F** Diameter measurement of C2C12 ASPA KO cells treated with NAA. Data are expressed as mean ± SD of n = 3 independent experiments (*p < 0.05 vs CTRL). **G** Representative images of C2C12 ASPA KO myotubes after staining with Oil Red O. Scale bars, 50 µm. (n = 3). **H** The ratio of MFI of cells untreated and treated with ATGListatin. Data are expressed as mean ± SD of n = 3 independent experiments (***p < 0.001 vs untreated). **I** Evaluation of extracellular lactate content in C2C12 ASPA KO cells. Lactate concentration was normalized on total proteins and data are expressed as mean ± SD of n = 3 independent experiments (*p < 0.05 vs CTRL Cas9).

To clarify the role of NAA catabolism in the metabolism of myotubes, we first characterized the expression of ASPA enzyme in the skeletal muscle. ASPA expression was comparable to that of the majority of human peripheral tissues, except adipose tissue and kidney, which exhibited very high expression levels [16] (Supplementary Fig. 4A). ASPA levels were higher in the oxidative muscle soleus than in the glycolytic muscle extensor digitorum longus (EDL) (Fig. 3A). Gene ontology (GO) analysis of genes that correlate with ASPA expression in muscle identified the GO terms linked to oxidative phosphorylation and fatty acid oxidation as the most significant ones (Fig. 3B). These results demonstrated a strong association between ASPA and oxidative metabolism in myofibers. Consistently, ASPA was upregulated during the glycolytic-to-oxidative switch we obtained after NAA treatment (Fig. 3C, D; Supplementary Fig. 4B).

Based on this evidence and to investigate whether NAA catabolism is required for the effects elicited by NAA treatment we generated ASPA KO C2C12 cells using CRISPR/Cas9 technique (Fig. 3E; Supplementary Fig. 4C). ASPA KO prevented NAA-induced changes in myotube diameter (Fig. 3F; Supplementary Fig. 4D), LDs content (Fig. 3G, H) and lactate extrusion (Fig. 3I). These results demonstrated that the phenotypic and metabolic effects of NAA were dependent on its catabolism by ASPA.

### Myotubes treated with NAA are more resistant to atrophic stimuli

**Fig. 4:**
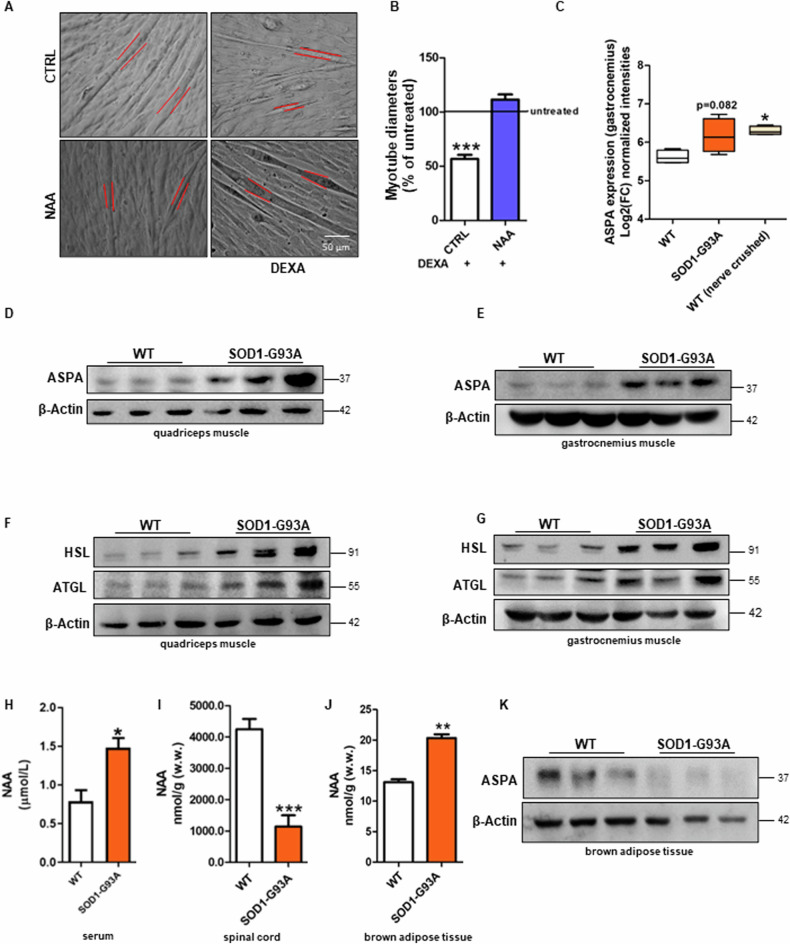
NAA restrains atrophic response in myotubes and ASPA is upregulated in muscles undergoing atrophy. C2C12 myotubes were treated with dexamethasone (DEXA) and after 48 h (**A**) bright field images of myotubes were taken. Scale bars, 50 µm (n = 3). **B** The diameter measurement was performed by ImageJ software. Data are expressed as mean ± SD of n = 3 independent experiments (*** p < 0.001 vs % of untreated). **C** Bioinformatic analysis of ASPA expression in gastrocnemius muscle of WT, SOD1-G93A and nerve-crushed mice. Data are expressed as Log2(fold change) ± SD (*p < *0.05 vs WT*). Western blot analysis of ASPA, HSL and ATGL levels on (**D**, **F**) quadriceps muscle and (**E**, **G**) gastrocnemius muscle of symptomatic SOD1-G93A and WT mice. β-Actin was used as a loading control. Determination of NAA concentration in **H** serum, **I** spinal cord and **J** BAT of SOD1-G93A and WT mice by HPLC. **K** Western blot analysis of ASPA on brown adipose tissue of symptomatic SOD1-G93A and WT mice. β-Actin was used as a loading control. The Western blots reported are representative of three independent experiments that gave similar results.

Oxidative fibers are known to be more resistant not only to physiological stimuli, such as exercise and fatigue, but also to atrophy [17]. Therefore, we monitored the response of NAA-treated myotubes challenged with atrophic stimuli. The pro-atrophic stimulus dexamethasone (DEXA) decreased the diameter of control cells, but not NAA-treated myotubes (Fig. 4A, B). Similar results were obtained using two additional atrophic stimuli, TNFα and cisplatin (Supplementary Fig. 4E, F).

As NAA catabolism was shown to be fundamental for the metabolic changes that lead to the glycolytic-to-oxidative fiber switch, we verified whether ASPA expression was modulated in in vivo models characterized by muscle atrophy. We observed that ASPA levels were increased in the gastrocnemius muscle of asymptomatic SOD1-G93A ALS mice and wild-type sciatic nerve-crushed mice, reaching statistical significance in this latter group (Fig. 4C). We then experimentally demonstrated the protein levels of ASPA were significantly upregulated in quadriceps and gastrocnemius of symptomatic SOD1-G93A ALS mice (Fig. 4D, E; Supplementary Fig. 5A, C) in parallel with high levels of the ATGL and HSL lipases (Fig. 4F, G; Supplementary Fig. 5B, D), which are consistent with the well-known metabolic switch from glycolytic to oxidative fibers in these muscles during ALS disease [18, 19].

ALS pathology is characterized by motor neuron degeneration and systemic hypermetabolism that we observed to be associated with BAT overactivation [20]. As these are the tissues that express the highest levels of the NAA-producing enzyme NAT8L, we extended our analysis on the NAA pathway to the spinal cord and BAT to characterize the sources of NAA that can feed muscles in SOD1-G93A ALS mice. After validating that serum NAA levels were increased in ALS mice (Fig. 4H), as previously described in humans, we demonstrated that this condition was coupled with a decreased content of the metabolite in the spinal cord (Fig. 4I) and no change in ASPA levels (Supplementary Fig. 5E, F). On the contrary, NAA was higher than controls in BAT of SOD1-G93A mice (Fig. 4J), whereas ASPA was decreased (Fig. 4K; Supplementary Fig. 5G).

## Discussion

This study highlighted for the first time the role of NAA catabolism in the metabolic adaptation of skeletal muscle. NAA administration augments lipid turnover in differentiated C2C12 myotubes, promoting both lipid synthesis and catabolism, as also observed in brown adipocytes overexpressing NAT8L [13]. NAA catabolism is necessary for the stimulation of lipid turnover, as no significant changes are detected in ASPA KO myotubes. The effect on lipogenesis is reasonably caused by the increase in acetate levels that can directly provide cytosolic acetyl-CoA as a substrate for the ACC enzyme. As concerns lipolysis, its activation may be the consequence of LDs accumulation to avoid that muscle cells may abnormally accumulate neutral lipids, a condition that is known to be detrimental for the muscle as demonstrated in several genetic diseases defective for lipid catabolic pathways [21]. We can also hypothesize that acetate itself may contribute to the activation of lipid catabolism as the enhancement of lipolysis was demonstrated in the skeletal muscle of mice fed with a supplementation of acetate [22]. A direct signaling role of NAA per se in both lipogenesis and lipolysis cannot be excluded.

The increase in lipid utilization is consistently supported by the stimulation of mitochondrial activity and biogenesis in C2C12 myotubes. Together these data and the upregulation of myoglobin demonstrated that the treatment with NAA promotes an oxidative phenotype in differentiated myotubes. Notably, this induction in the oxidative pathway was coupled to a decrease in anaerobic glycolysis after NAA treatment. This result was reverted when a lipolysis inhibitor or ASPA KO cells were used, further demonstrating that NAA catabolism and consequent ATGL activation are the drivers of the metabolic shift boosting mitochondrial metabolism while inactivating glycolytic flux.

Besides metabolism, NAA treatment promoted other phenotypic changes typical of oxidative myofibers, including the upregulation of MHC7b and MHC IIa and the increase in protein turnover (increased protein synthesis and autophagic/proteasomal degradation). Consistently, the glycolytic-to oxidative switch described in ALS skeletal muscle [10] and in the spinal and bulbar muscular atrophy [23], is also associated with the upregulation of oxidative-specific MHC. On the contrary, this change in MHC isotypes was not observed when the oxidative muscle soleus shifted towards a glycolytic phenotype in mice defective for mitochondrial long-chain fatty acid oxidation [24].

In this scenario, the upregulation of PGC1α following NAA treatment seems to be fundamental as it is the master player in the glycolytic-to-oxidative switch of muscle cells promoting mitochondrial biogenesis, oxidative metabolism and the expression of type I MHC isoforms [25, 26]. Moreover, PGC1α is also known to protect skeletal muscle from atrophy, indirectly, by buffering ROS [27] and, directly, by suppressing atrophy-specific gene transcription [17]. This is consistent with the slight reduction of *FBXO32* expression after NAA treatment and the inhibitory action of NAA on the diameter of myotubes challenged with different atrophic stimuli.

Beyond ALS, the glycolytic-to-oxidative switch of the skeletal muscle is frequently associated with muscle atrophy also in other physiological and pathological conditions. Age-associated skeletal muscle atrophy in glycolytic fibers is linked to a decrease in mitochondrial enzyme expression while type I fibers seem to be initially protected due to the higher content of mitochondria [19]. Analogously, the decrease of PGC1α expression and oxidative fiber content has a detrimental effect on muscle homeostasis leading to muscle atrophy in Huntington’s disease [28]. Conversely, oxidative myofibers are largely exposed to atrophy after muscle disuse and shift toward glycolysis [29]. Therefore, the metabolic changes occurring in such conditions are likely to be an adaptation to limit skeletal muscle dysfunction, most likely because oxidative fibers are more resistant than glycolytic ones. In the context of pathological conditions linked to motor neuron damage such as ALS, in which NAA accumulation occurs in blood, the oxidative phenotype promoted by NAA treatment and the inhibition of atrophic response suggest that NAA functions as a protection/compensation mechanism, forcing ALS muscles towards a more resistant oxidative phenotype. This is also supported by the upregulation of ASPA enzyme we described in ALS muscles undergoing the glycolytic-to-oxidative switch and in denervated muscles.

An important aspect to consider is the source of NAA that can impact skeletal muscle metabolism. Differently from neurons and brown adipocytes, muscle cells show very low or no expression of NAT8L. A major interaction between NAA and skeletal muscle can occur in those pathological states showing increased blood concentration of NAA and muscle alterations, including ALS. A direct release of NAA at the level of the neuromuscular junction can occur following the degeneration of motoneurons. We cannot exclude that local production of NAA in muscle can also derive from immune cells that can be present in both homeostatic and injured skeletal muscle as well as from the muscle fibro-adipogenic progenitors that can transdifferentiate in brown/beige adipocytes to recover muscle degeneration [30]. In the SOD1-G93A mouse model, we observed an increase of NAA levels in serum while a reduction in the spinal cord that is consistent with the degenerative process of the tissue. Therefore, neurodegeneration is likely to represent the primary mechanisms involved in NAA release due to the very high abundance of this metabolite in neurons. Nevertheless, the well-known cross-talk between BAT and skeletal muscle has to be also mentioned given that a hyperactivation of BAT in ALS mouse models was demonstrated [20]. The increased production of NAA in ALS BAT, which is coupled with its impaired intracellular catabolism due to ASPA downregulation, may partially contribute to NAA release during the disease course as a consequence of muscle metabolic reprogramming.

Overall, our results highlighted an unpredicted role of NAA as a key driver of muscle metabolic identity that, fostering lipid turnover and oxidative metabolism, may safeguard myotubes from physiological or disease-associated atrophic conditions.

## Supplementary information

Supplemental Materials

Original Western Blot Images

## Data Availability

Bioinformatic analyses of ASPA expression in EDL and soleus muscle were performed on microarray data and the raw data is available in the GEO database (accession number GSE23244). The expression levels of ASPA in gastrocnemius of SOD1-G93A and nerve-crushed mice are available from the GSE16362 dataset. ASPA expression in the different tissues was analyzed from the Genotype-Tissue Expression dataset (GTex; https://www.gtexportal.org). Gene ontology (GO) enrichment analysis of genes co-expressed with ASPA has been performed using the Database for Annotation, Visualization and Integrated Discovery (DAVID) web tool (https://david.ncifcrf.gov). The original Western blot data are provided in Supplementary Materials (Original Western blots).
