# Supplementary material for: N-acetylaspartate promotes glycolytic-to-oxidative fiber-type switch and resistance to atrophic stimuli in myotubes
Source: Cell Death Dis. 2024 Sep 19;15(9):686. doi: 10.1038/s41419-024-07047-0 (PMC11413391; doi:10.1038/s41419-024-07047-0)
Supplement: Supplementary file 1 — Supplemental Materials [file 41419_2024_7047_MOESM1_ESM.docx]

**Supplementary Materials for:**

**N-acetylaspartate promotes glycolytic-to-oxidative fiber-type switch and resistance to atrophic stimuli in myotubes**

Serena Castelli^1^, Enrico Desideri^2^, Leonardo Laureti^3^, Federica Felice^3^, Angela De Cristofaro^3^, Silvia Scaricamazza^4^, Giacomo Lazzarino^5^, Maria Rosa Ciriolo^1,3,*^ and Fabio Ciccarone^1,3,*,a^

^1^ IRCCS San Raffaele Roma, 00166 Rome, Italy

^2^ Department of Human Sciences and Promotion of the Quality of Life, San Raffaele Open University, 00166 Rome, Italy

^3^Department of Biology, University of Rome “Tor Vergata”, 00133 Rome, Italy

^4^ IRCCS Fondazione Santa Lucia, 00179 Rome, Italy

^5^ UniCamillus-Saint Camillus International University of Health and Medical Sciences, 00131 Rome, Italy

^*^ These authors contributed equally to this work

^a^ Corresponding author. E-mail address: fabio.ciccarone@uniroma2.it (F. Ciccarone)

**Materials and methods**

**Materials**

β-mercaptoethanol (444203), 2-propanol (34863), Albumin (A3782), Dimethyl sulfoxide (DMSO, 154938), EDTA (E6758), Glycerol (G7893), Glycine (G8898), Hydrazine (216046), Nicotinamide adenine dinucleotide hydrate (NAD+) (N7004), Oil Red-O (ORO) (O0625), Formalin solution neutral buffered 10% (HT5012), Sodium chloride (S8776), Sodium deoxycholate (D6750), Sodium orthovanadate (S6508), Sodium pyrophosphate tetrabasic decahydrate (30411), Trichloroacetic acid (TCA) (T6399) and Triton X-100 (T9284) were from Sigma-Aldrich, St. Louis, MO, USA. Goat anti-mouse (172-1011) and anti-rabbit (172-1019) IgG (H+L)-horseradish peroxidase-conjugated, TRIS-base (1610716) and Sodium Dodecyl Sulfate (SDS) (161-0300) were from Bio-Rad Laboratories, Hercules, CA, USA. L-lactate dehydrogenase (LDH) was from Roche Applied Science, Penzberg, Germany. DTT (281) and protease inhibitors cocktail from AMRESCO, Boise, Idaho. JC-1 (420200) is from Calbiochem, San Diego, CA, USA. TEMED (110189) and TRItidy G (T95424) are from PanReac AppliChem, Milano, Italy.

**Cell lines, treatments and animals.**

The cell line of murine myoblasts C2C12 was purchased from the American Type Culture Collection (ATCC). Cells were grown in Dulbecco’s modified Eagle’s medium (DMEM) 4.5 g/L glucose, supplemented with 10% fetal bovine serum, 10 U/ml penicillin/streptomycin, 2 mM L-glutamine, 1 mM sodium pyruvate and 10 mM HEPES pH 7.0. All the cell culture media and supplements were purchased from Euroclone, Milan, Italy. Mycoplasma test was routinely performed, according to protocols from our laboratory. Cells were cultured at 37 °C and 5% CO2; for all the experiments, they were plated at a 5 × 10^4^ cells/mL density on wells covered by 0.1 g/mL of gelatin derived from bovine skin (G9391, Sigma Aldrich). After 24 h, C2C12 cells’ differentiation in myotubes was induced by a specific differentiation medium consisting of DMEM containing 4.5 g/L of glucose, supplemented with 2% of horse serum (HS), 2 mM of L-glutamine and 10 U/mL penicillin/streptomycin solution, 1 mM sodium pyruvate and 10 mM HEPES pH 7.0, all provided by EuroClone. The differentiation was induced for 7 days, changing the medium every day, except where differently indicated. The treatment with NAA (Sigma Aldrich, 00920) was added after 5 days of differentiation and was left for 48 h, until the end of differentiation. For the experiments performed after 15 days of differentiation, NAA was added from the 13^th^ day until the end of the experiment. NAA was dissolved in water, adjusting pH to 7.0 with NaOH (VWR International, Radnor, PA, USA).

Cells were treated with 25 μM ATGListatin (SML1075, Sigma-Aldrich), 0.1 μM MG132 (474791, Sigma-Aldrich), 20 mM NH_4_Cl (A9434, Sigma-Aldrich) and 50 μM leupeptin (SAE0153, Sigma-Aldrich), 100 μM Dexamethasone (D4902, Sigma-Aldrich), 100 μM TNFalfa (T7539, Sigma-Aldrich), 50 μM Cisplatin (232120, Sigma-Aldrich) for 48 h; 1 μM puromycin (P8833, Sigma-Aldrich) for 2 h. 400 μM oleic acid (O1008, Sigma Aldrich) was administered 16 h before NAA treatment and was dissolved in bovine serum albumin (BSA) 0.05% solution, heated at 37° C for 30 min before use.

All animals were approved by the Animal Welfare Office, Department of Public Health and Veterinary, Nutrition and Food Safety, General Management of Animal Care and Veterinary Drugs of the Italian Ministry of Health (protocol number 931/2017/PR) and performed following the European Guidelines for the use of animals in research (2010/63/EU) and the requirements of Italian laws (D.L. 26/2014). Animals were fed ad libitum and kept in virus/antigen-free conditions with a light/dark cycle of 12 h at constant temperature and humidity. SOD1-G93A mice (B6.Cg-Tg(SOD1 G93A)1Gur/J) were purchased from The Jackson Laboratory (Bar Harbor, ME, USA). Hemizygous SOD1-G93A males and C57BL/6 females were crossbred, and progeny were genotyped by PCR. Disease onset was evaluated by hanging grid test; serum was from 10-weeks mice while BAT and spinal cord from 20-22 weeks-old mice. Before sacrifice, mice were anaesthetized with Rompum (xylazine, 20 mg/ml, 0.5 ml/kg Bayer, Milan, Italy) plus Zoletil (tiletamine and zolazepam, 100 mg/ml, 0.5 ml/kg; Virbac, Milan, Italy).

**Knock-Out production by CRISPR/Cas9 technology.**

The generation of ASPA and ATGL Knock-Out (KO) C2C12 cells was performed the CRISPR/Cas9 technology. The Cas9 plasmid (AddGene, Teddington, UK) was composed of constitutive Chicken β-actin promoter (Cbh) and puromycin resistance gene (PuroR) for selection of the transfected cells. For ASPA KO, the Cas9 enzyme was directed by gRNA, obtained from the following guides: ASPA 1, forward 5́-CACCGAGTGCAACCCATGTTAGAAG-3 and reverse 5́-AAACCTTCTAACATGGGTTGCACTC; ASPA 2 forward 5́-CACCGAGTGCAACCCATGTTAGAAG-3 and reverse 5-AAACCTTCTAACATGGGTTGCACTC-3. For ATGL KO, the following guides were used: ATGL 1, forward 5-CACCGAGAGGCGGTAGAGATTGCGA-3 and reverse 5- AAACTCGCAATCTCTACCGCCTCTC-3; ATGL 2, forward 5-CACCGGCAGGAGGCCACGCCAATG-3 and reverse 5-AAACCATTGGCGTGGCCTCCTGCC-3. For control, only the Cas9 plasmid was transfected.

After 24 h plating cells were transfected with the abovementioned plasmids for 24 h by the PEI reagent, according to the manufacturer’s instructions. The selection was performed with 2 µg/mL puromycin for 72 h. then, the fresh medium was replaced, and the cells were left to grow. The success of the knock-out was tested by Western blot.

**Western blot analysis.**

Cells were resuspended in lysis buffer (50 mM Tris-HCl, pH 7.4, 150 mM NaCl, 1 mM EDTA, 1% Triton X-100, 0.5% sodium deoxycholate, 0.1% SDS, 10 mM sodium fluoride, 5 mM Sodium Pyrophosphate, 2 mM Sodium Orthovanadate) complemented with protease inhibitor cocktail (AMRESCO). The protein concentration was determined by the Lowry method, before performing electrophoresis by SDS-PAGE.

The following primary antibodies were used: ACC1 (#4190, Cell Signaling Technology, Beverly, MA, USA), pACC1 Ser79 (#3661, Cell Signaling Technology), Acetylated-lysine (32268, Santa Cruz Biotechnology, Dallas, TX, USA), ACO2 (NBP1-90264, NovusBio, Centennial, Colorado), ASPA (NBP1-31754, NovusBio), ATGL (#2438S, Cell Signaling Technology), BNIP3 (B7931, Sigma-Aldrich), HK-2 (H00003099-M01, Abnova, Taipei, Taiwan), HSL (#4107S, Cell Signaling Technology), LC3 (L7543, Sigma-Aldrich), MHC (MF20, DSHB), mTOR (#2972, Cell Signaling Technology), p-mTOR Ser2448 (#2971, Cell Signaling Technology), NRF1 (sc-515360, Santa Crux Biotechnology), PGC1α (ST1202, Sigma-Aldrich), p-p70s6k Thr389 (#9205, Cell Signaling Technology), Puromycin (3RH11, Kerafast, Boston, MA, USA), TFAM (166965, Santa Cruz Biotechnology), ubiquitine (#3933, Cell Signaling Technology) α-Actinin (#3134S, Cell Signaling Technology), β-Actin (#4970S, Cell Signaling Technology). After the incubation with specific secondary antibodies (Bio-Rad), the signal was detected using a Fluorchem Imaging System (Alpha Innotech, San Leandro, USA), incubating membranes with LiteAblot TURBO (EuroClone). Fluorchem Imaging System (Alpha Innotech) was used to perform densitometry analyses.

**Microscopy analyses.**

Live cells were treated with 2.5 µM JC-1 (420200, Calbiochem) for 30 min at 37 °C. Delta Vision Restoration Microscopy System (Applied Precision, Issaquah, WA) equipped with an Olympus IX70 fluorescence microscope (Olympus Italia, Segrate, Milano, Italy) was used to acquire fluorescent images of cells. The 535 nm filter was used to acquire green fluorescence (monomers) and a 590 nm filter was used for red fluorescence (J-aggregates). The evaluation of the ratio between red and green fluorescence was performed by ImageJ software. For the analyses of myotube diameters and the fusion index, the same microscope was used to obtain bright field images. ImageJ software was exploited to measure myotube diameter and to count nuclei in each myotube. In bright field images, red lines were used to mark representative myotubes.

**Extracellular lactate assay.**

Cell medium (500 µl) was precipitated with 250 µl of 30% trichloroacetic acid (TCA). Media were stored at -20° for at least 1 h and then centrifuged at 14 000×g for 20 min at 4°C. 10 µl of supernatant were incubated with 290 µl of reaction buffer composed by 0.2 M glycine, 0.2 M hydrazine buffer pH 9.2 with freshly added 0.6 mg/ml NAD+ and 17 U/ml LDH enzyme for 30 min at 37°C. The amount of lactate was quantified by measuring NADH absorbance at 340 nm using an Eppendorf BioSpectrometer. Relative absorbance values were converted to lactate concentration using NADH extinction coefficient at 340 nm of 6,220 M-1cm-1. Concentrations were normalized on total proteins in each sample.

**Complex I activity assay.**

Cells were resuspended in ice-cold lysis buffer (25 mM HEPS-Na, 2 mM MgCl, 1 mM EDTA and 1 mM EGTA) complemented with protease inhibitor cocktail (AMRESCO). Samples were frozen and thawed 3 times and then centrifuged at 14.000xg for 10 min at 4 °C. Ten µg of proteins were incubated with the reaction buffer (Potassium phosphate buffer 0.5 M pH 7.5, BSA 50 mg/mL, KCN 10 mM, NADH 5 mM w/without Rotenone 1 mM). After added 4mM CoQ1, absorbance was recorded at 340 nm for 2 min at 30 °C. To assess rotenone sensitive NADH-dehydrogenase activity, we performed parallel reactions in presence and absence of rotenone (complex I inhibitor). Data were expressed nmol of NADH produced in 1 min per 1 µg of proteins.

**Oil Red-O staining.**

Cells were washed in PBS and fixed in formalin solution neutral buffered 10% (4% paraformaldehyde containing) for 10 min. After PBS washes, cells were incubated for 5 min with 60% isopropanol and stained with the Oil Red-O (filtered 6:4 dilution of a pre-filtered 0.35% Oil Red-O stock solution in 100% isopropanol) for 10 min. Oil Red-O was removed and di-deionized water was used to wash cells. Delta Vision Restoration Microscopy System (Applied Precision) equipped with an Olympus IX70 fluorescence microscope (Olympus) was used to acquire fluorescent images of cells. The used excitation and detection wavelengths were 560 nm and 690 nm, respectively.

**RNA isolation and Real-Time qPCR**

RNA extraction was performed by using TRItidy G (PanReac AppliChem) according to the manufacturer’s instructions. Synthesis of cDNA was obtained from 1 μg of total RNA by using iScript™ Reverse Transcription Supermix for RT-qPCR (Bio-Rad), and RT-qPCR reaction was performed by using the iTaq Universal SYBR Green Supermix (Bio-Rad) on QuantStudio™ 3 real-time PCR System (Thermo Fisher Scientific).

Primers were designed and tested with primer-BLAST (NCBI) and purchased from Sigma-Aldrich. The following murine primer pairs were used: *Myoglobin,* forward: 5’-CTGTTTAAGACTCACCCTGAGAC-3’, reverse: 5’-GGTGCAACCATGCTTCTCTA-3’, *MyH7b,* forward: 5’-CTCAAGCGGGAGAACAAGAATC-3’, reverse: 5’-CTGAGGCTGACCTGGTCTGTAA-3’, *MyH2,* forward: 5’-AGTCCCAGGTCAACAAGCTG-3’, reverse: 5’-GCATGACCAAAGGTTTCACA-3’, *MyH4,* forward: 5’-AGTCCCAGGTCAACAAGCTG-3’, reverse: 5’-TTTCTCCTGTCACCTCTCAACA-3’, *FBXO32,* forward: 5’-GCGACCTTCCCCAACGCCTG-3’, reverse: 5’-GGCGACCGGGACAAGAGTGG-3’,  *TRIM63,* forward: 5’-AGGGGCTACCTTCCTCTCAAGTG-3’, reverse: 5’-TCTTCCCCAGCTGGCAGCCC-3’, *ACTB*, forward: 5’-CACACCCGCCACCAGTTCGC-3’, reverse: 5’-TTGCACATGCCGGAGCCGTT-3’. The relative mRNA levels were determined by using the 2*^−ΔΔCt^* method. The fold changes were relative to the control; the normalization was performed to the internal standard *ACTB*.

**HPLC analysis**

HPLC analyses for NAA were carried out using a Hypersil C-18, 250 × 4.6 mm, 5 μm particle size column (Thermo Fisher Scientific, Rodano, Milan, Italy) in a Surveyor HPLC apparatus (Thermo Fisher Scientific, Rodano, Milan, Italy) equipped with a highly sensitive 5 cm light-path flow cell diode array UV detector, setup for acquisition between 200 and 550 nm wavelengths. Both columns used were provided with their own guard columns. Data acquisition and analysis were performed using the ChromQuest^®^ software package provided by the HPLC manufacturer.  NAA was quantified at 206 nm wavelength in deproteinized extracts by matching retention times, peak areas and absorption spectra of those of ultrapure standards.

**Bioinformatic analyses.**

ASPA expression in EDL and soleus muscles was assessed using expression data from the GSE23244 (10 samples) dataset. The expression levels of ASPA in gastrocnemius of SOD1-G93A and nerve-crushed mice were analysed by exploiting data from the GSE16362 dataset (4 samples). All datasets abovementioned were derived from Gene Expression Omnibus (GEO; [http://www.ncbi.nlm.nih.gov/geo](http://www.ncbi.nlm.nih.gov/geo)). ASPA expression in the different tissues was analyzed from the Genotype-Tissue Expression dataset (GTex; <https://www.gtexportal.org>). Gene ontology (GO) enrichment analysis of genes co-expressed with ASPA has been performed using the Database for Annotation, Visualization and Integrated Discovery (DAVID) web tool (https://david.ncifcrf.gov). Statistical analysis was performed using the method indicated in the figure legend.

**Data analysis.**

The results are presented as means ± SD of data derived from at least 3 independent experiments. Student t-test for comparison was used for statistical analyses of only two variables and one-way ANOVA with post hoc Tukey for multiple comparisons. The GraphPad Prism 7 software was used. Comparisons were considered statistically significant at p≤0.05 (*), very statistically significant at p≤0.01 (**) and extremely statistically significant at p≤0.001 (***).


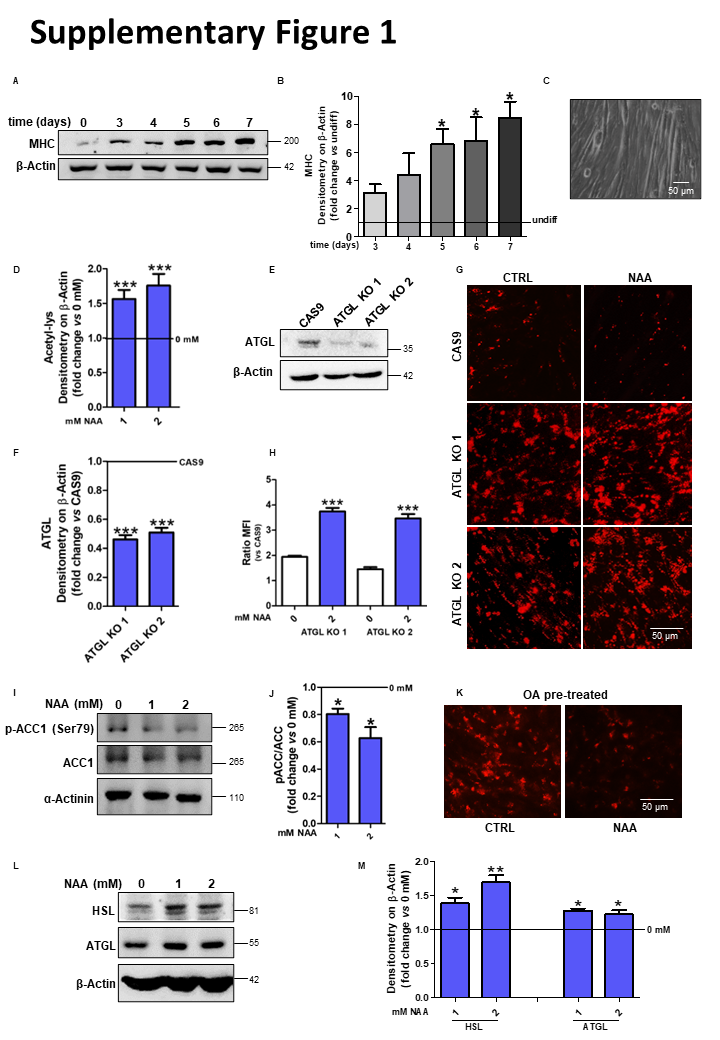


**Supplementary Figure 1.** (**A**) Western blot analysis of Myosin heavy-chain (MHC) levels was performed on C2C12 cells after the indicated time of differentiation. β-Actin was used as a loading control. (**B**) Densitometric analysis of MHC levels (shown in Figure 1A). Data are expressed as mean ± SD of n=3 independent experiments (** p<0.05 vs* undifferentiated). (**C**) Representative bright field image of myotubes after 5 days of differentiation. Scale bars, 50 µm. (n=3). (**D**) Densitometric analyses of acetyl-lysine levels of differentiated C2C12 myotubes treated with NAA (shown in Figure 1B). Data are expressed as mean ± SD of n=3 independent experiments (**** p<0.001 vs* 0 mM). (**E**) Representative western blot and (**F**) densitometric analyses of ATGL expression in ATGL WT and KO C2C12 cells. β-Actin was used as a loading control. Data are expressed as mean ± SD of n=3 independent experiments (**** p<0.001 vs* Cas9). (**G**) Representative images of differentiated ATGL KO C2C12 myotubes treated with NAA and stained with Oil Red O. Scale bars, 50 µm. (n=3). (**H**) Quantification of MFI of cells indicated in (F). Data are expressed as the mean ratio of MFI ± SD of n=3 independent experiments (*** p <0.001 vs Cas9). (**I**) Western blot and (**J**) densitometric analysis of p-ACC1 (Ser79) and ACC1. (**K**) Representative images of differentiated C2C12 myotubes stained with Oil Red O after pre-loading with oleic acid (OA) and treatment with NAA. Scale bars, 50 µm. (n=3). ). (**L**) Western blot and (**M**) densitometric analysis of HSL and ATGL.


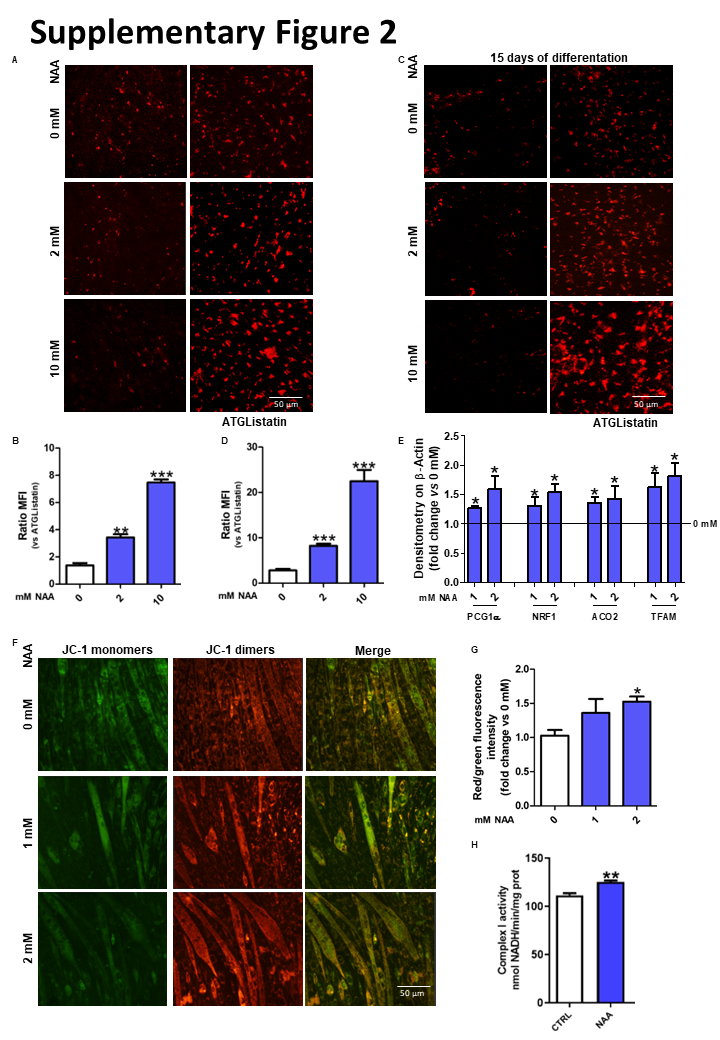


**Supplementary Figure 2.** Representative images of (**A**) 7-days and (**C**) 15-days differentiated C2C12 myotubes treated with NAA for the last 48 hours, with/without ATGListatin and stained with Oil Red O. Scale bars, 50 µm. (n=3).  (B and D) Quantification of MFI of cells indicated in (A and C), respectively. Data are expressed as the mean ratio of MFI ± SD of n=3 independent experiments (*** p <0.001 *vs* ATGListatin). (**E**) Densitometric analysis of western blot of PGC1α, NRF1, ACO2 and TFAM levels (shown in Figure 1E). Data are expressed as mean ± SD of n=3 independent experiments (** p<0.05 vs* 0 mM and untreated). (**F**) Fluorescence microscopy analysis of C2C12 myotubes stained with JC-1. Scale bars, 50 µm. (n=3) (**G**) Quantification of the ratio between red and green fluorescence intensity was performed with the ImageJ software. Data are expressed as mean ± SD of n=3 independent experiments (* p<0.05 vs 0 mM). (**H**) Spectrophotometric analysis of Complex I activity. Data are expressed as mean ± SD of n=3 independent experiments (* p<0.05 vs CTRL).


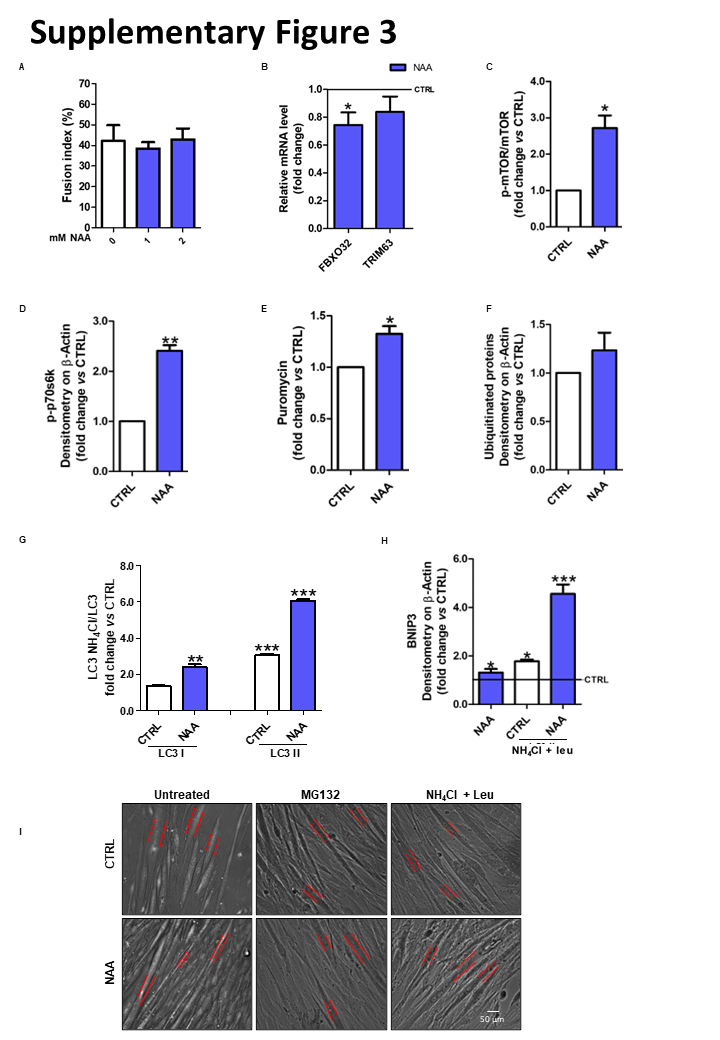


**Supplementary Figure 3.** (**A**) Fusion index of differentiated C2C12 myotubes, measured as the number of nuclei located within MHC-positive myotubes divided by the total number of nuclei, evaluated with the ImageJ software. Data are expressed as mean ± SD of n=3 independent experiments (** p<0.05 vs* % of 0 mM). (**B**) RT-qPCR analysis of *FBXO32* (Atrogin) and *TRIM63* (Murf1*)* in differentiated C2C12 myotubes. *ACTB* was used as a reference control. Data are shown as fold change ± SD of n=3 independent experiments (* *p <0.05 vs* CTRL). CTRL was represented by a straight line in the bar graph. (**C-E**) Densitometric analysis of (**C**) pmTOR/mTOR, (**D**) p-p70s6k (both shown in Figure 2C) and (**E**) puromycin (shown in Figure 2D) in differentiated C2C12 myotubes. Data are expressed as mean ± SD of n=3 independent experiments (** p<0.05,* ** *p<0.01 vs* CTRL). (**F**) Densitometric analysis of ubiquitinated proteins (shown in Figure 2E). Data are expressed as mean ± SD of n=3 independent experiments (** p<0.05 vs* CTRL). Densitometric analysis of (**G**) BNIP3 and (**H**) LC3I and LC3II (both shown in Figure 2F). NH_4_Cl/leupeptin was used as co-treatment with NAA. Data are expressed as mean ± SD of n=3 independent experiments (** p<0.05,* ** *p<0.01, *** p<0.001 vs* CTRL). (**I**) Bright-field images of myotubes treated as in (D) and (E) Scale bars, 50 µm. (n=3).


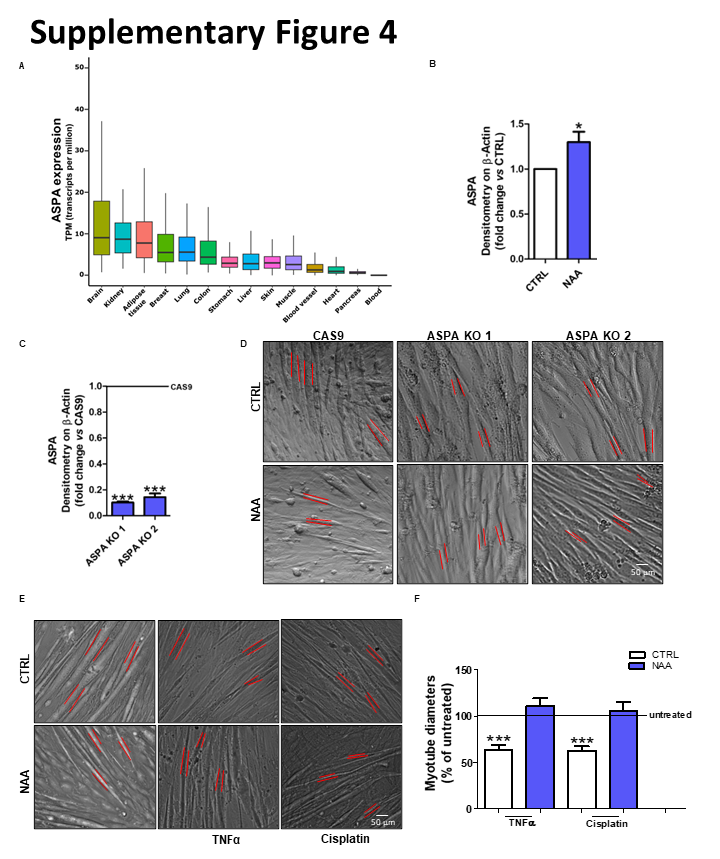


**Supplementary Figure 4.** (**A**) Bioinformatic analysis of ASPA expression in different tissues based on the GTEX database. Data are shown as box and whiskers plot of transcript per million. (**B-C**) Densitometric analysis of ASPA levels in NAA-treated (shown in Figure 3D) (**B**) and ASPA KO (shown in Figure 3E) (**C**) of differentiated C2C12 cells. Data are expressed as mean ± SD of n=3 independent experiments (** p<0.05 vs* CTRL). (**D**) Bright-field images of ASPA WT and KO C2C12 myotubes. Scale bars, 50 µm. (n=3). (**E**) Bright-field images of C2C12 myotubes co-treated with NAA and either TNFα or cisplatin. Scale bars, 50 µm. (n=3). (**F**) The diameter measurement was performed with the ImageJ software. Data are expressed as mean ± SD of n=3 independent experiments (**** p<0.001 vs % of* untreated).


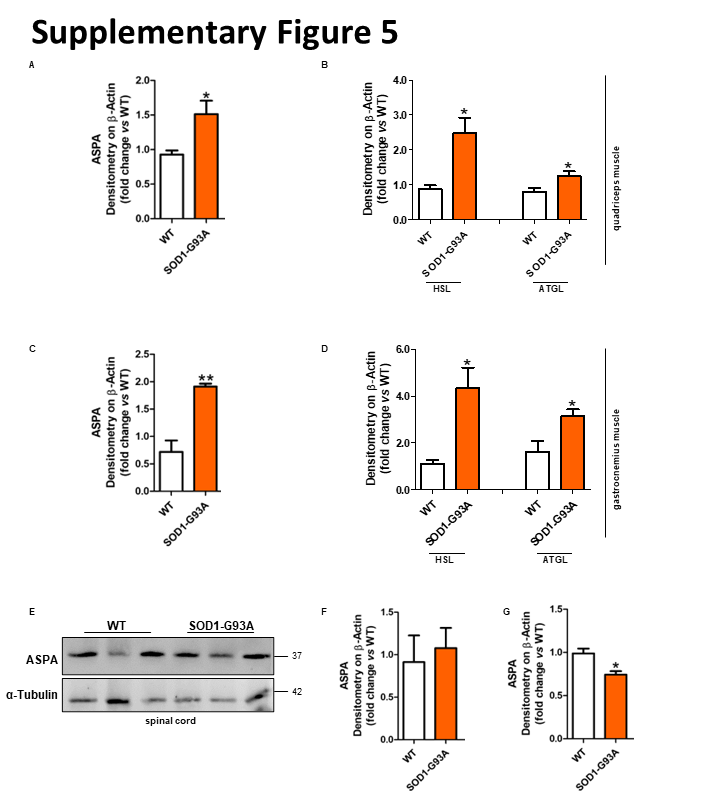


**Supplementary Figure 5. (A-B)** Densitometric analysis of (**A**) ASPA (shown in Figure 4D), (**B**) HSL and ATGL levels in the quadriceps (shown in Figure 4F) (**A**) of WT and SOD1-G93A mice. (**C-D**) Densitometric analysis of ASPA (shown in Figure 4E) (**C**) and HSL and ATGL (shown in Figure 4G) (**D**) levels in the gastrocnemius of WT and SOD1-G93A mice. (**E-F**) Western blot (E) and densitometric (**F**) analyses of ASPA expression in the spinal cord of WT and SOD1-G93A mice. β-Actin was used as a loading control. (n=3). (**G**) Densitometric analysis of ASPA expression in the brown adipose tissue of WT and SOD1-G93A mice (shown in Figure 4J). Data are expressed as mean ± SD of n=3 independent experiments (** p<0.05,* *** p<0.01 vs* WT).
