# Supplementary material for: N-acetylaspartate promotes glycolytic-to-oxidative fiber-type switch and resistance to atrophic stimuli in myotubes
Source: Cell Death Dis. 2024 Sep 19;15(9):686. doi: 10.1038/s41419-024-07047-0 (PMC11413391; doi:10.1038/s41419-024-07047-0)
Supplement: Supplementary file 2 — Original Western Blot Images [file 41419_2024_7047_MOESM2_ESM.pptx]

## Slide 1
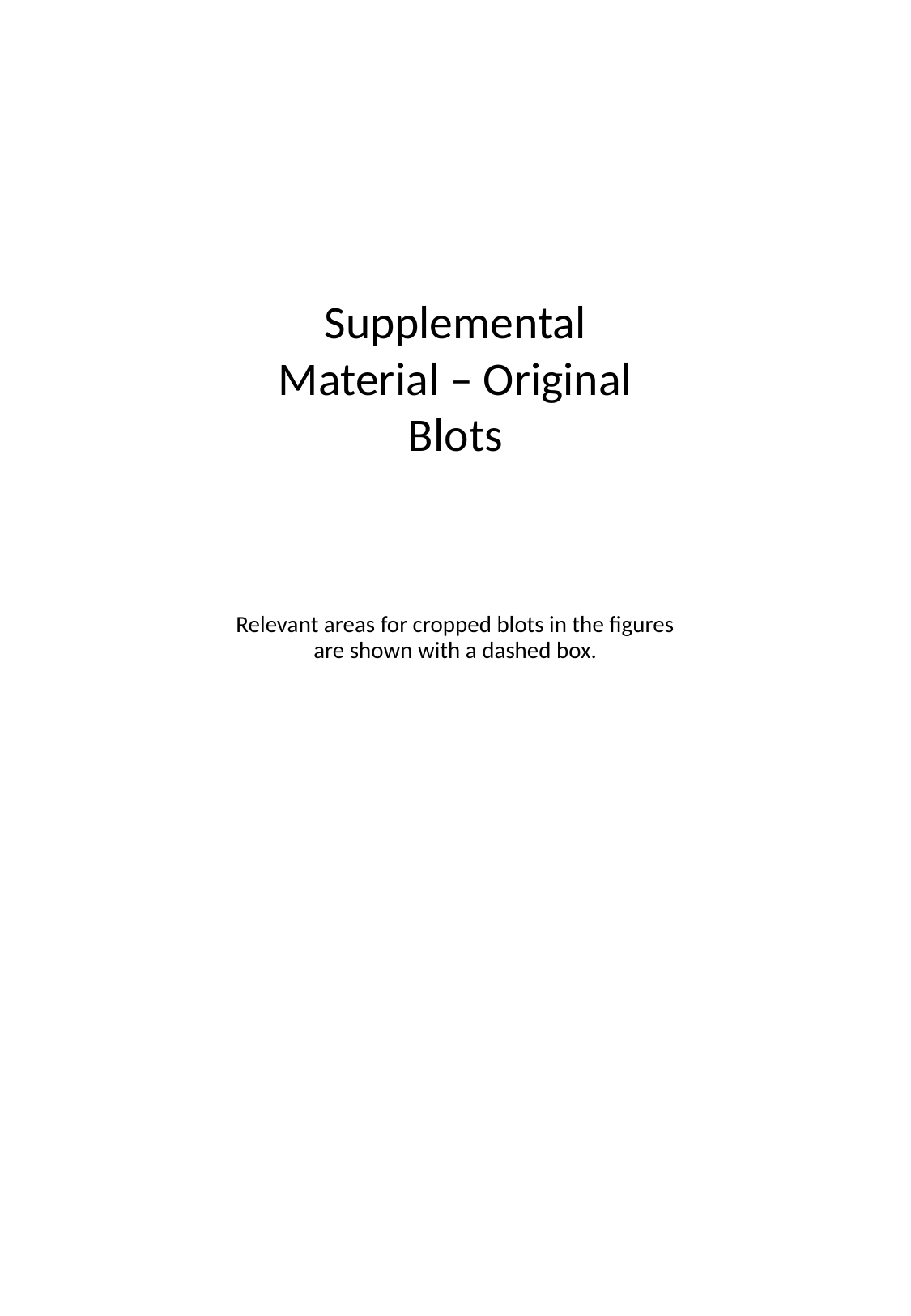

Supplemental Material – Original Blots
Relevant areas for cropped blots in the figures are shown with a dashed box.

## Slide 2
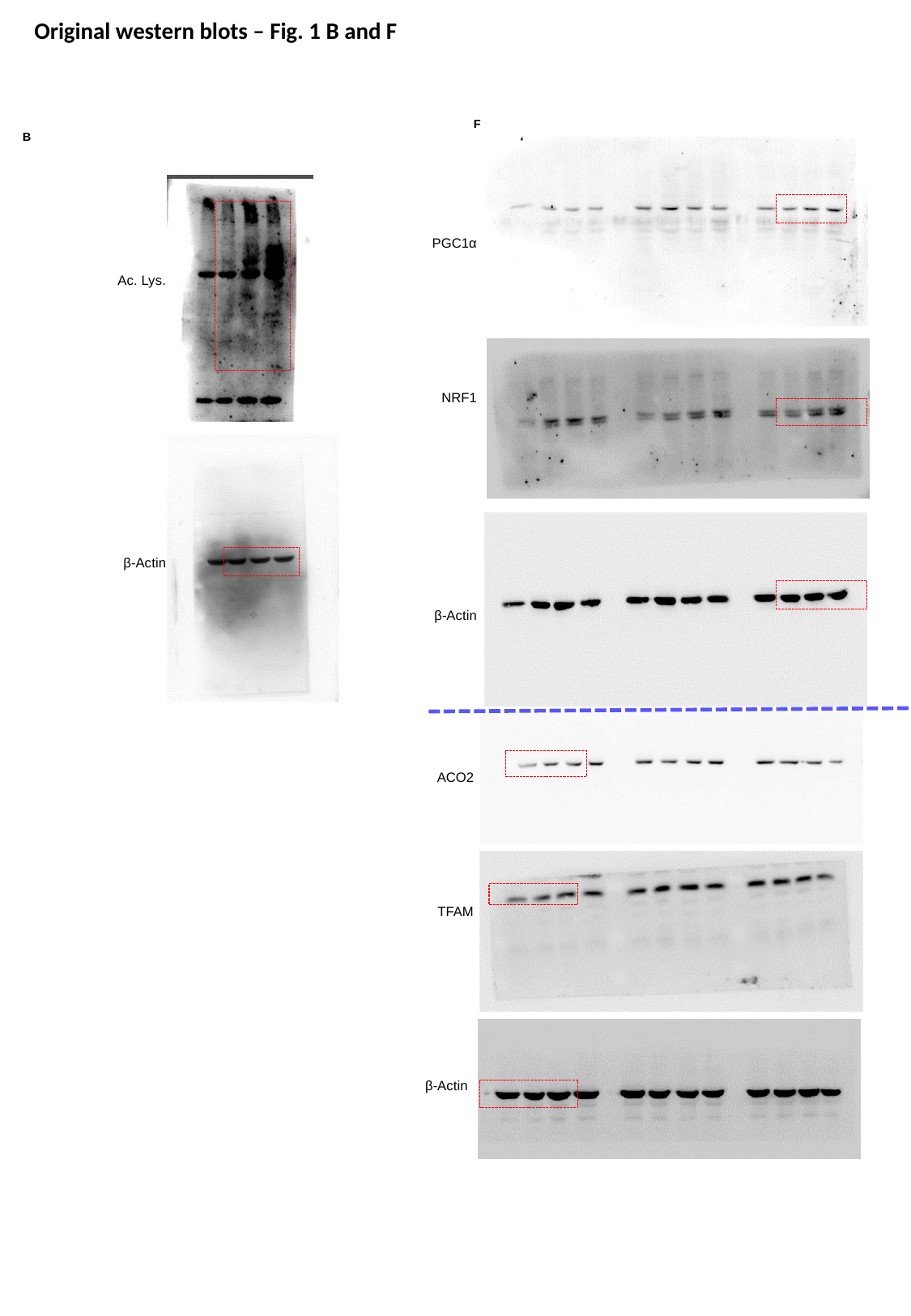

Original western blots – Fig. 1 B and F
F
B
PGC1α
Ac. Lys.
NRF1
β-Actin
β-Actin
ACO2
TFAM
β-Actin

## Slide 3
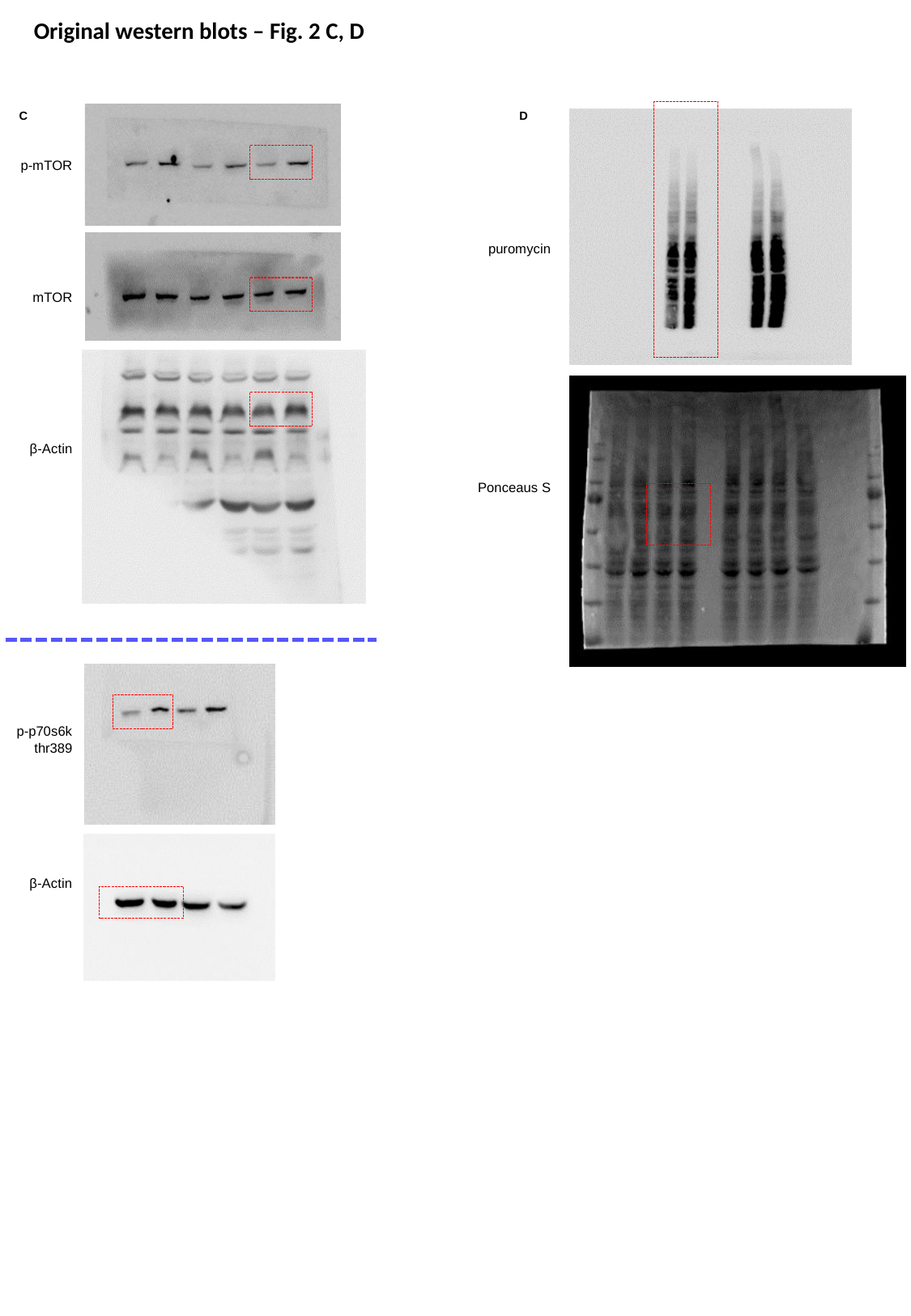

Original western blots – Fig. 2 C, D
C
D
p-mTOR
puromycin
mTOR
β-Actin
Ponceaus S
p-p70s6k
 thr389
β-Actin

## Slide 4
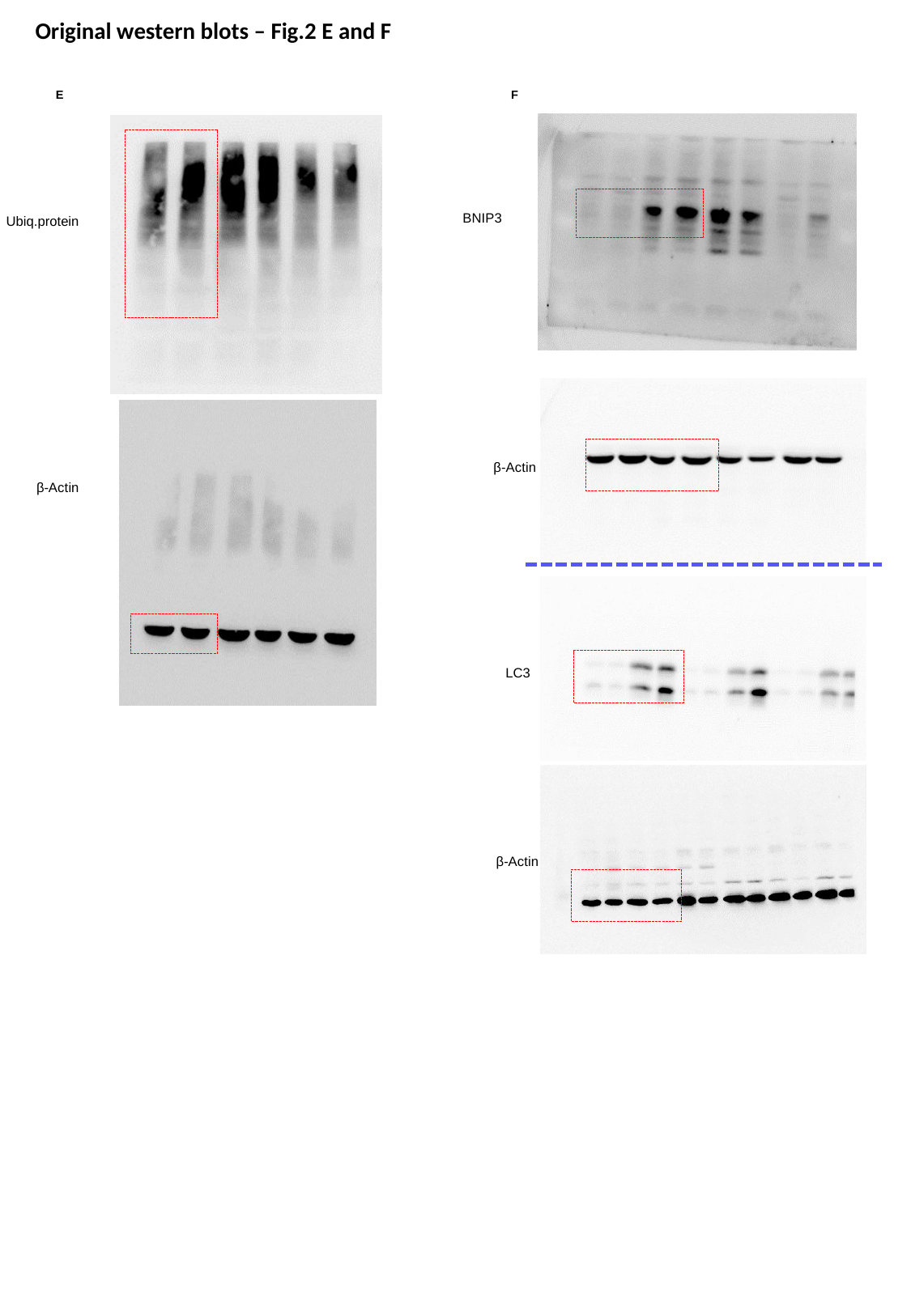

Original western blots – Fig.2 E and F
E
F
BNIP3
Ubiq.protein
β-Actin
β-Actin
LC3
β-Actin

## Slide 5
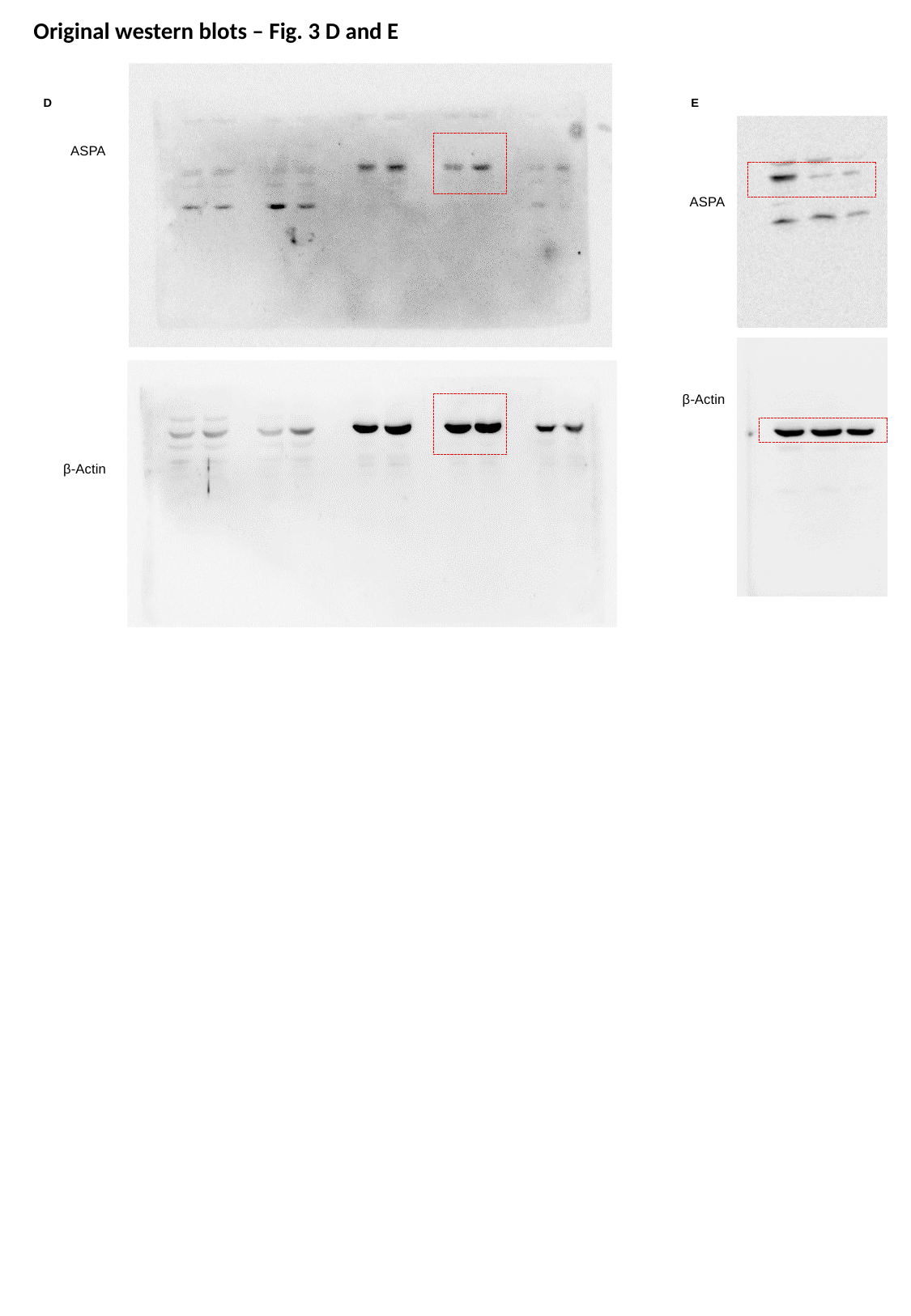

Original western blots – Fig. 3 D and E
D
E
ASPA
ASPA
β-Actin
β-Actin

## Slide 6
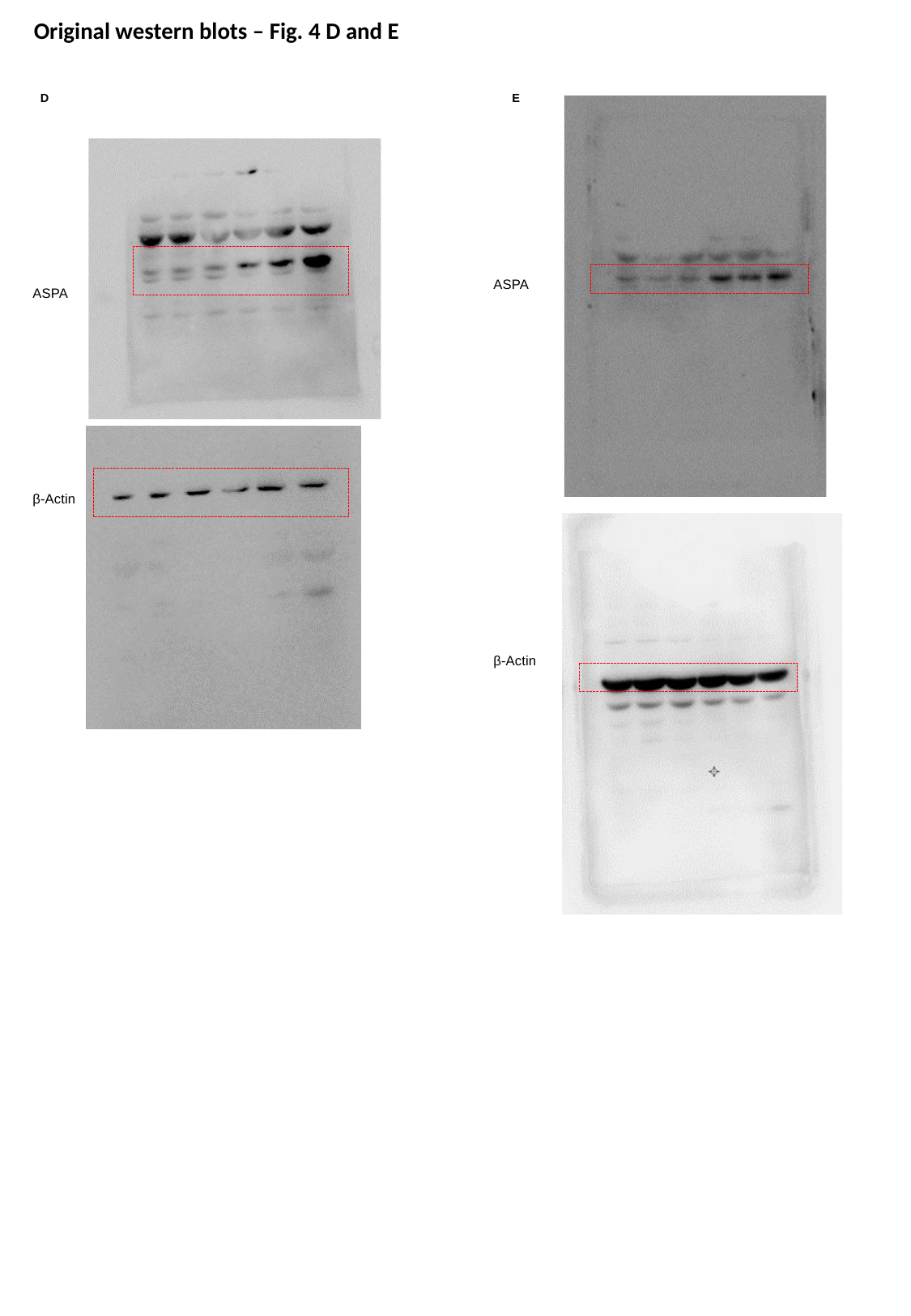

Original western blots – Fig. 4 D and E
D
E
ASPA
ASPA
β-Actin
β-Actin

## Slide 7
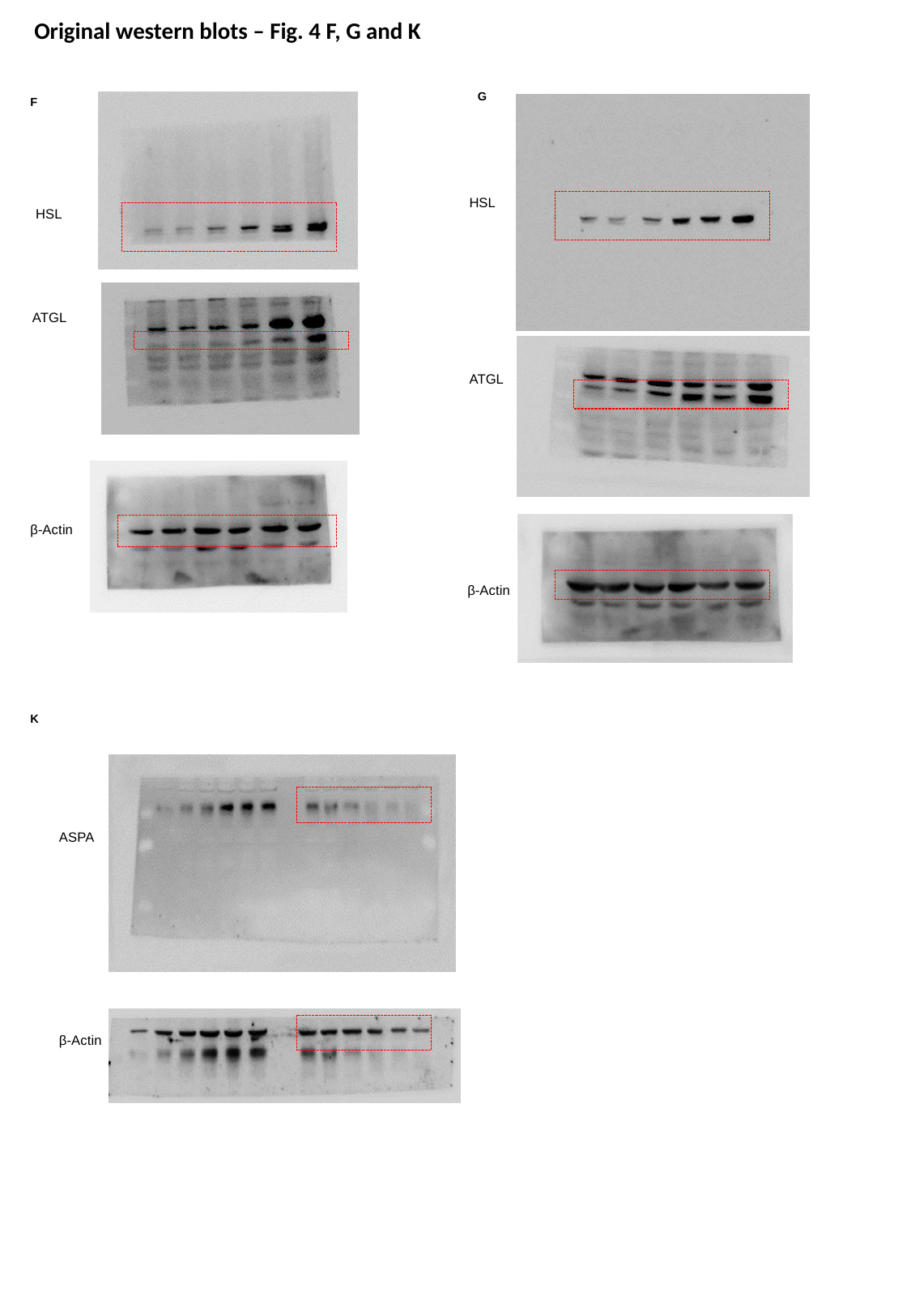

Original western blots – Fig. 4 F, G and K
G
F
HSL
HSL
ATGL
ATGL
β-Actin
β-Actin
K
ASPA
β-Actin

## Slide 8
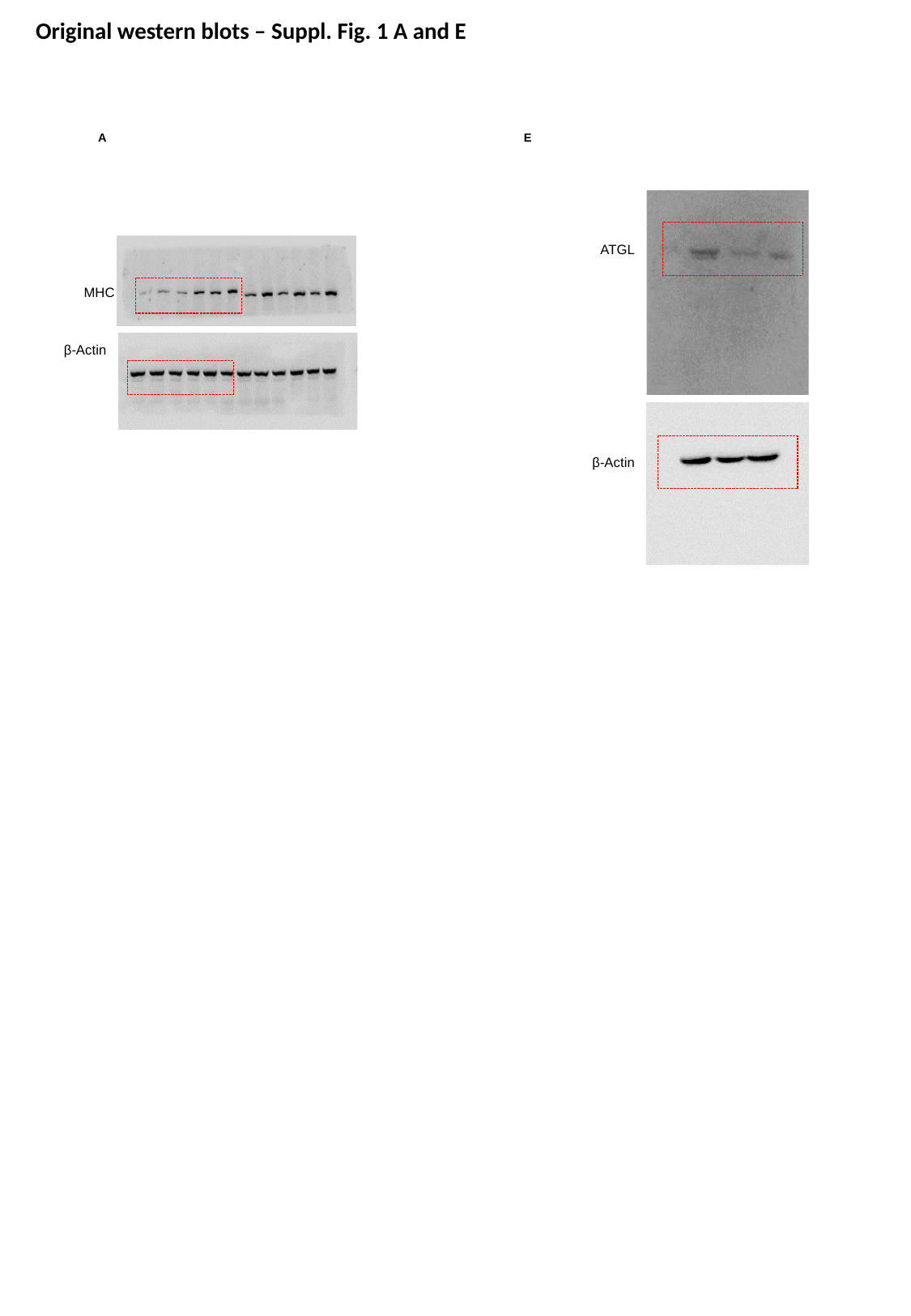

Original western blots – Suppl. Fig. 1 A and E
A
E
ATGL
MHC
β-Actin
β-Actin

## Slide 9
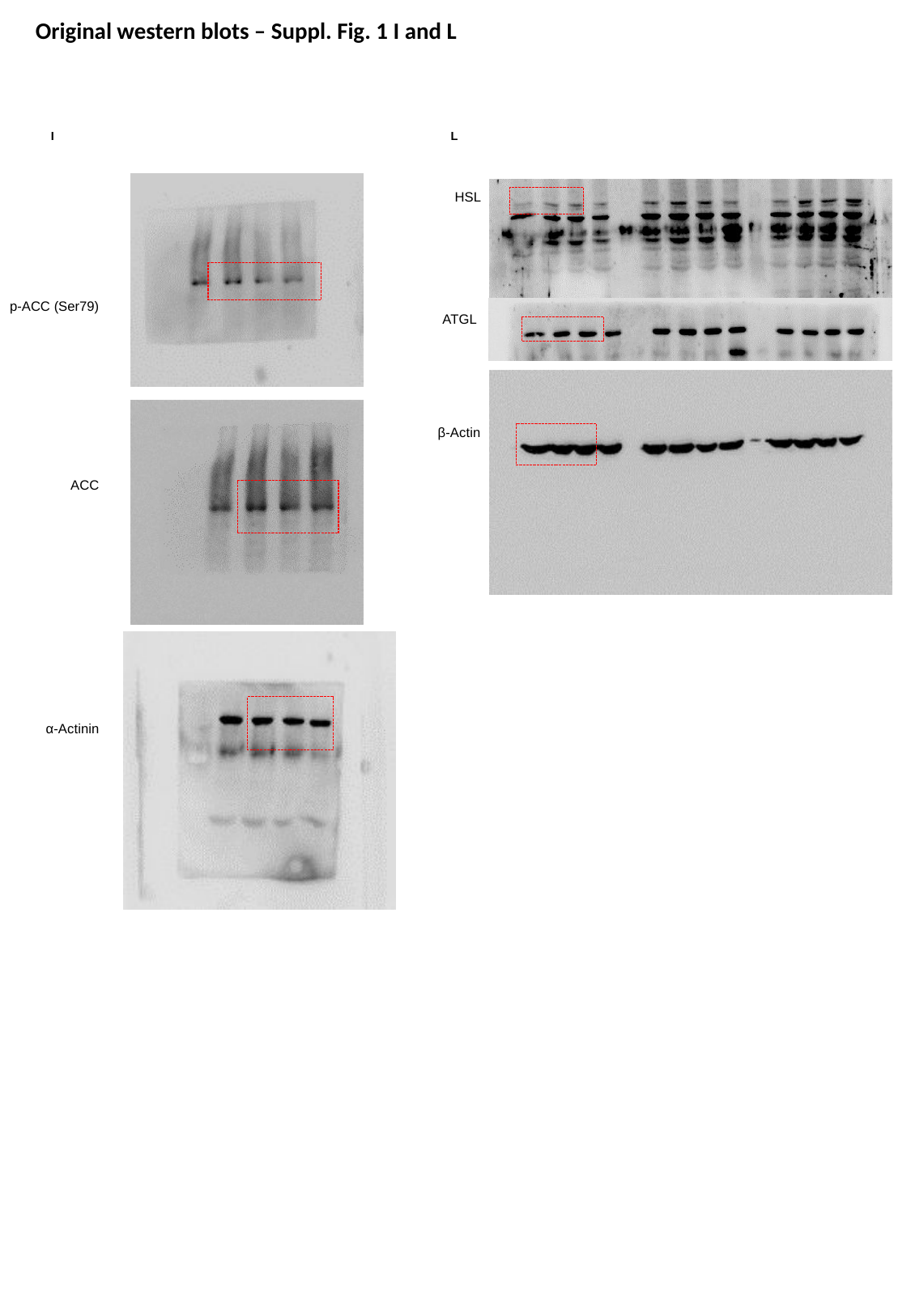

Original western blots – Suppl. Fig. 1 I and L
I
L
HSL
p-ACC (Ser79)
ATGL
β-Actin
ACC
α-Actinin

## Slide 10
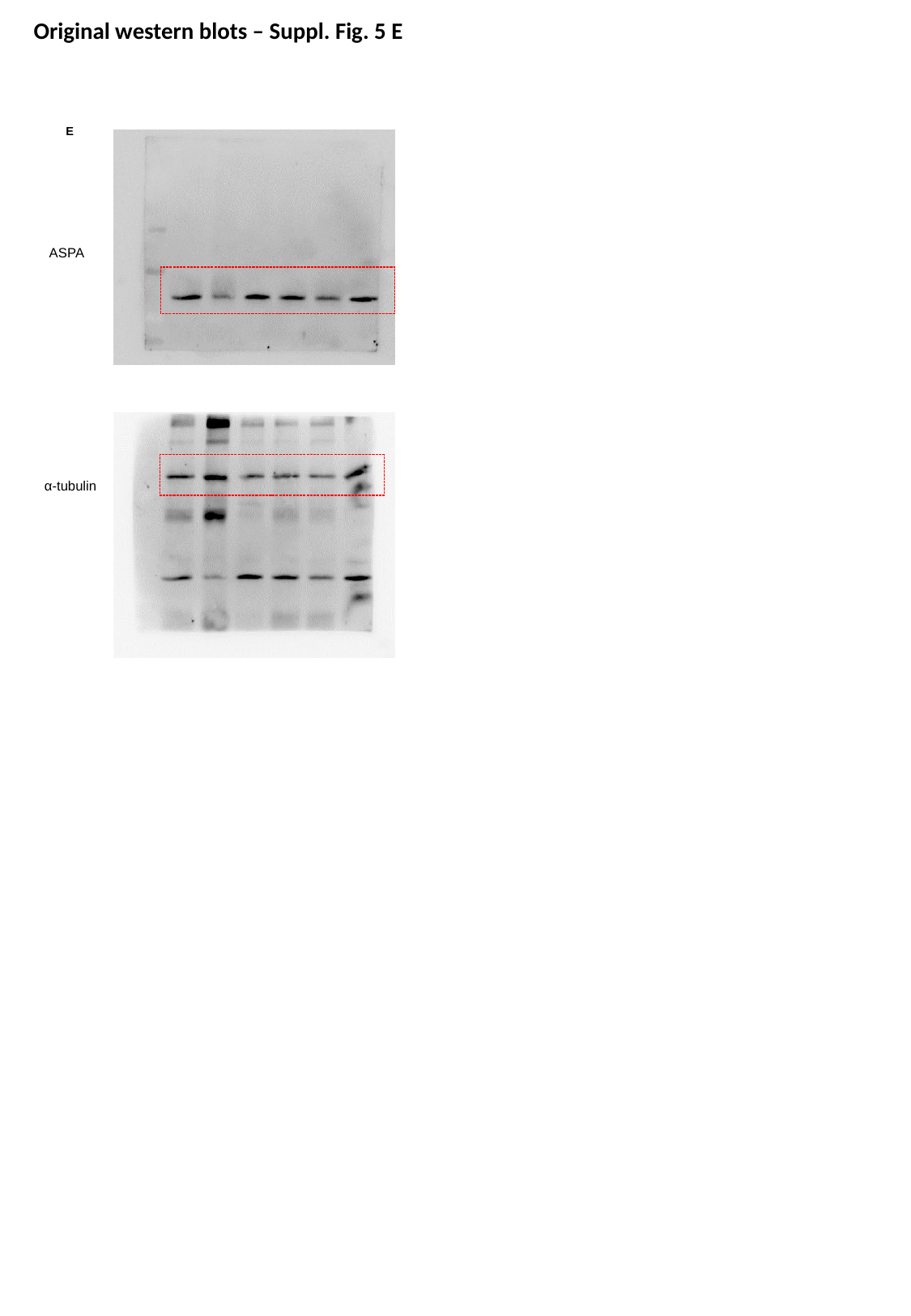

Original western blots – Suppl. Fig. 5 E
E
ASPA
α-tubulin
